# Estimating the Acute Health Impacts of Fire‐Originated PM_2.5_ Exposure During the 2017 California Wildfires: Sensitivity to Choices of Inputs

**DOI:** 10.1029/2021GH000414

**Published:** 2021-07-01

**Authors:** Stephanie E. Cleland, Marc L. Serre, Ana G. Rappold, J. Jason West

**Affiliations:** ^1^ Department of Environmental Sciences and Engineering Gillings School of Global Public Health University of North Carolina at Chapel Hill Chapel Hill NC USA; ^2^ Oak Ridge Institute for Science and Education at the Center for Public Health and Environmental Assessment Office of Research and Development United States Environmental Protection Agency Research Triangle Park NC USA; ^3^ Center for Public Health and Environmental Assessment Office of Research and Development United States Environmental Protection Agency Research Triangle Park NC USA

**Keywords:** air pollution, fine particulate matter, wildfire smoke, health impact assessment, population exposure, health impacts

## Abstract

Exposure to wildfire smoke increases the risk of respiratory and cardiovascular hospital admissions. Health impact assessments, used to inform decision‐making processes, characterize the health impacts of environmental exposures by combining preexisting epidemiological concentration–response functions (CRFs) with estimates of exposure. These two key inputs influence the magnitude and uncertainty of the health impacts estimated, but for wildfire‐related impact assessments the extent of their impact is largely unknown. We first estimated the number of respiratory, cardiovascular, and asthma hospital admissions attributable to fire‐originated PM_2.5_ exposure in central California during the October 2017 wildfires, using Monte Carlo simulations to quantify uncertainty with respect to the exposure and epidemiological inputs. We next conducted sensitivity analyses, comparing four estimates of fire‐originated PM_2.5_ and two CRFs, wildfire and nonwildfire specific, to understand their impact on the estimation of excess admissions and sources of uncertainty. We estimate the fires accounted for an excess 240 (95% CI: 114, 404) respiratory, 68 (95% CI: −10, 159) cardiovascular, and 45 (95% CI: 18, 81) asthma hospital admissions, with 56% of admissions occurring in the Bay Area. Although differences between impact assessment methods are not statistically significant, the admissions estimates' magnitude is particularly sensitive to the CRF specified while the uncertainty is most sensitive to estimates of fire‐originated PM_2.5_. Not accounting for the exposure surface's uncertainty leads to an underestimation of the uncertainty of the health impacts estimated. Employing context‐specific CRFs and using accurate exposure estimates that combine multiple data sets generates more certain estimates of the acute health impacts of wildfires.

## Key Points

- PM_2.5_ exposure during the 2017 California fires accounted for an estimated 308 excess respiratory and cardiovascular hospital admissions
- Health impact assessments, used to inform decision‐making processes, are sensitive to the exposure and epidemiologic inputs specified
- Accurate exposure estimates and context‐specific health risk functions estimate fire‐attributable health impacts with greater certainty

## 1 Introduction

In recent years, California has experienced a number of devastating wildfires. These fires adversely impact air quality both locally and regionally for long periods of time (Reisen et al., 2015), elevating concentrations of fine particulate matter (PM_2.5_) to levels potentially hazardous to human health. The October 2017 northern California wildfires resulted in record high PM_2.5_ concentrations, with daily average concentrations greater than 190 μg/m^3^, dangerously exceeding the 24‐h PM_2.5_ standard of 35 μg/m^3^ and exposing large populations to unhealthy air (Bay Area Air Quality Management District, 2019; Cleland et al., 2020). Exposure to fire‐originated PM_2.5_ can cause a variety of adverse health outcomes, with substantial evidence for increased risk of respiratory morbidity and growing evidence for increased risk of cardiovascular morbidity and all‐cause mortality (Jaffe et al., 2020; Liu et al., 2015; Reid et al., 2016). As the frequency, intensity, and spread of wildfires are likely to increase due to climate change (Boegelsack et al., 2018; Liu et al., 2016; Spracklen et al., 2009; Yue et al., 2013), increasing population‐level exposure to fire‐originated PM_2.5_ (Ford et al., 2018), it is necessary to improve upon existing methods to better identify and quantify the health impacts of wildfire smoke exposure.

Two primary ways of evaluating the health impacts of wildfire smoke are epidemiological studies, which aim to evaluate health risk and health burden based on health outcome data, and health impact assessments, which aim to evaluate the overall health impact using health risk estimates from prior epidemiological studies. There is a growing body of epidemiological evidence that short‐term smoke exposure increases respiratory and cardiovascular hospital and emergency department (ED) admissions (Borchers‐Arriagada et al., 2019; Deflorio‐Barker et al., 2019; Delfino et al., 2009; Gan et al., 2020; Haikerwal et al., 2015; Liu et al., 2017; Rappold et al., 2012). While essential for understanding the public health implications of fire events, epidemiological risk estimates require detailed health outcome data to be available, which can lag in years, limiting the ability to inform decision making during or just prior to wildfire seasons. In contrast, impact assessments rely on preexisting concentration–response functions (CRFs) with estimated health risks from epidemiological studies, which when combined with current information on pollutant exposure, baseline health incidence, and population density can estimate the total number of hospital and/or ED admissions attributable to wildfire smoke exposure (Borchers‐Arriagada, Palmer, Bowman, Morgan, et al., 2020; Borchers‐Arriagada, Palmer, Bowman, Williamson, et al., 2020; Bowman et al., 2019; Broome et al., 2016; Fann et al., 2013, 2018; Jiang & Yoo, 2019; Johnston et al., 2021; Matz et al., 2020; Rittmaster et al., 2006). Each of the inputs into a health impact assessment contributes to the overall precision and accuracy of the wildfire smoke‐attributable health impacts estimated, but their influence is largely unknown and rarely quantified.

In prior assessments, PM_2.5_ exposure during wildfires has been estimated using three primary data sets: monitoring station observations, chemical transport models (CTMs), and satellite observations (Borchers‐Arriagada, Palmer, Bowman, Morgan, et al., 2020; Borchers‐Arriagada, Palmer, Bowman, Williamson, et al., 2020; Bowman et al., 2019; Broome et al., 2016; Fann et al., 2013, 2018; Jiang & Yoo, 2019; Johnston et al., 2021; Matz et al., 2020; Rittmaster et al., 2006). Fire‐originated PM_2.5_ has been isolated using either monitoring station observations over the same geographic region during a nonfire period (Borchers‐Arriagada, Palmer, Bowman, Morgan, et al., 2020; Borchers‐Arriagada, Palmer, Bowman, Williamson, et al., 2020; Bowman et al., 2019; Broome et al., 2016) or a CTM run during the fire period without fire emissions (Fann et al., 2013, 2018; Jiang & Yoo, 2019; Matz et al., 2020). The CRFs selected also vary, with many assessments using CRFs for nonwildfire, ambient PM_2.5_ (Borchers‐Arriagada, Palmer, Bowman, Morgan, et al., 2020; Borchers‐Arriagada, Palmer, Bowman, Williamson, et al., 2020; Bowman et al., 2019; Broome et al., 2016; Fann et al., 2013; Johnston et al., 2021; Matz et al., 2020) and only some employing wildfire‐specific (WF) CRFs (Fann et al., 2018; Jiang & Yoo, 2019). Further, the majority of prior wildfire‐focused health impact assessments did not account for uncertainty beyond the uncertainty of the CRF and only a select few considered more than one exposure surface or CRF. As wildfires become an increasingly prevalent public health issue, it is important to identify which elements of the impact assessment framework have the most influence on the magnitude and uncertainty of the health impacts estimated in order to understand how best to conduct these assessments moving forward.

Here, we calculate the number of daily excess respiratory, cardiovascular, and asthma hospital admissions attributable to fire‐originated PM_2.5_ exposure during the October 2017 California wildfires and characterize how these estimates change with alternative choices of CRFs and smoke exposure surfaces. More specifically, we evaluate how different epidemiological health risk and PM_2.5_ exposure estimates and their associated uncertainty influence both the number of attributable admissions estimated and their confidence bounds. To our knowledge, this is the first systematic assessment of how choices of inputs for health impact assessments affect estimates of the acute health impacts of wildfire smoke exposure. By showing how different sources of uncertain data influence health impact estimates for wildfires, our findings can help strengthen future impact assessments and inform public health decision‐making processes before, during, and after fire events.

## 2 Data and Methods

### 2.1 PM_2.5_ Exposure Data

We used three different total PM_2.5_ and two different background PM_2.5_ exposure surfaces to generate four estimates of daily average, ground‐level fire‐originated PM_2.5_ at a 1‐km resolution. In addition to the exposure surface used in our base case impact assessment, three alternative approaches were used in a sensitivity analysis, described in detail below, two evaluating different estimates of total PM_2.5_ and one evaluating a different background PM_2.5_ estimate. The three estimates of total PM_2.5_ exposure, which were previously developed (Cleland et al., 2020), are: space–time (s/t) kriging of monitoring station observations, using the Bayesian Maximum Entropy (BME) Framework (BME kriging); bias‐corrected Community Multiscale Air Quality (CMAQ) model output, using the Constant Air Quality Model Performance (CAMP) method (CC‐CMAQ); and a BME data fusion of observations with CC‐CMAQ and satellite‐derived concentrations (BME data fusion). The two estimates of background PM_2.5_ exposure are: the percent of PM_2.5_ attributable to background emissions, obtained from CMAQ output with and without fire emissions (CMAQ percent attributable) and BME kriging of observations from October 2016 (October 2016). The four estimates of fire‐originated PM_2.5_ are: BME data fusion with CMAQ percent attributable; BME kriging with CMAQ percent attributable; CC‐CMAQ with CMAQ percent attributable; and BME data fusion with October 2016.

To develop the BME kriging and BME data fusion exposure surfaces, daily average PM_2.5_ observations were used as input. Observations were obtained from permanent Federal Reference Methods (FRM)/Federal Equivalent Methods (FEM) and temporary non‐FRM/FEM monitoring stations across California for October 1–31, 2017. For the CC‐CMAQ and BME data fusion exposure surfaces, we used CMAQ simulations, a widely used CTM developed by the United States (U.S.) Environmental Protection Agency (EPA), as input. The CMAQ simulations, which used estimates of fire emissions derived from satellite observations and estimates of all other anthropogenic and natural emissions, provided estimates of daily average PM_2.5_ at a 4‐km resolution in central California for October 3–20, 2017. The third input into the BME data fusion was satellite‐derived PM_2.5_ concentrations, which we generated by converting aerosol optical depth (AOD) observations to PM_2.5_ estimates using a day‐specific linear mixed effects model. The 3‐km resolution AOD data were acquired from the Moderate Resolution Imaging Spectroradiometer (MODIS) Terra Satellite across California for October 1–31, 2017.

CC‐CMAQ was generated using the CAMP method, which improves the accuracy of CTM output by applying a bias correction that accounts for the nonlinear, heteroscedastic relationship between observed and modeled concentrations (de Nazelle et al., 2010; Reyes et al., 2017). CC‐CMAQ estimates both the bias‐corrected PM_2.5_ concentration and its associated variance. The BME kriging and BME data fusion surfaces were generated using the BME framework, which uses modern s/t geostatistics to accurately estimate PM_2.5_ and measures of associated uncertainty at unmonitored locations by combining information on the trends and variability of the data with information on concentrations at a set of known s/t locations (Christakos, 1990; Christakos et al., 2002; Serre & Christakos, 1999). To produce estimates of ground‐level PM_2.5_ concentrations and their associated variance, BME s/t kriging was used to interpolate monitoring observations and BME data fusion was used to fuse observations with both CC‐CMAQ and the satellite‐derived concentrations. Compared to CC‐CMAQ and BME kriging, BME data fusion provides the most accurate and precise estimates of ground‐level PM_2.5_ in the fire‐affected region and period (Cleland et al., 2020). While no prior acute health impact assessment has used the fusion of observed, modeled, and satellite‐derived PM_2.5_ concentrations to assess smoke exposure, both monitoring station observations and CMAQ output, standalone and in combination, have been used as exposure estimates (Borchers‐Arriagada, Palmer, Bowman, Morgan, et al., 2020; Borchers‐Arriagada, Palmer, Bowman, Williamson, et al., 2020; Bowman et al., 2019; Fann et al., 2013, 2018; Jiang & Yoo, 2019; Johnston et al., 2021).

To estimate background PM_2.5_, concentrations without emissions from the October 2017 fires, and isolate the fire‐originated component from the total PM_2.5_, we used two different data sets: modeled concentrations when CMAQ is run without fire emissions and FRM/FEM monitoring station observations from October 2016, when California fire activity was identified to be low (Text S1; Figures S1 and S2). For the CMAQ percent attributable approach, we used the ratio between the CMAQ output with only nonfire emissions and with both fire and nonfire emissions to identify the percent of PM_2.5_ attributable to background emissions at any s/t location. Using this approach, fire‐originated PM_2.5,_ ΔX(s,t), is calculated as $\Delta X\left(s,\;t\right)=X\left(s,\;t\right)-X_{NF\_CMAQ}\left(s,\;t\right)$, where X(s,t) is the total PM_2.5_ concentration at location set s on day t, obtained from one of the three exposure estimates described above, and $X_{NF\_CMAQ}\left(s,\;t\right)=X\left(s,\;t\right)\times CMAQ_{back}\left(s,\;t\right)/CMAQ_{fire}\left(s,\;t\right)$, where $CMAQ_{back}$ and $CMAQ_{fire}$ are the CMAQ model output without and with fire emissions, respectively. While prior impact assessments have used CTMs run without fire emissions to determine background PM_2.5_ during fires (Fann et al., 2013, 2018; Matz et al., 2020), only one has used it in a relative manner (Jiang & Yoo, 2019). For the October 2016 approach, we used BME kriging to spatially interpolate the monthly average of monitoring stations observations across California during October 2016. Using this approach, fire‐originated PM_2.5_, ΔX(s,t), is calculated as $\Delta X\left(s,\;t\right)=X\left(s,\;t\right)-X_{NF\_2016}\left(s\right)$, where $X_{NF\_2016}\left(s\right)$ is the estimated October 2016 monthly average PM_2.5_ concentration at location set s. We selected October 2016 because no large fires occurred in that time period and the seasonal meteorological conditions and nonfire emissions were likely similar to those during October 2017. Similar approaches have been used to identify fire‐originated PM_2.5_ in previous impact assessments (Borchers‐Arriagada, Palmer, Bowman, Morgan, et al., 2020; Borchers‐Arriagada, Palmer, Bowman, Williamson, et al., 2020; Bowman et al., 2019). In both approaches, to avoid negative concentrations, ΔX(s,t) is set to zero if $X_{NF\_2016}\left(s\right)$ or $X_{NF\_CMAQ}\left(s,\;t\right)$ exceeds X(s,t).

### 2.2 Concentration–Response Functions

We considered two types of CRFs: WF CRFs and ambient, nonwildfire‐specific (NF) CRFs. The WF CRFs are from an epidemiological study of the 2003 southern California wildfires, which found a 2.8% (95% confidence interval [CI]: 1.4, 4.1) increase in respiratory, a 0.8% (95% CI: −0.1, 1.8) increase in cardiovascular, and a 4.8% (95% CI: 2.1, 7.6) increase in asthma hospital admissions per 10 µg/m^3^ increase in 2‐day average PM_2.5_ (Delfino et al., 2009). Risk coefficients were also calculated for age subgroups for respiratory and asthma admissions and for sex subgroups for asthma admissions. These CRFs have been used in previous health impact assessments of wildfire smoke (Fann et al., 2018; Jiang & Yoo, 2019).

The ambient, NF CRFs are from an epidemiological study of 26 U.S. counties between 2000 and 2003, which found a 2.07% (95% CI: 1.20, 2.95) increase in respiratory and 1.89% (95% CI: 1.34, 2.45) increase in cardiovascular hospital admissions per 10 µg/m^3^ increase in 2‐day average ambient PM_2.5_ (Zanobetti et al., 2009). These CRFs have been used in a previous health impact assessment of wildfire smoke exposure (Fann et al., 2018).

### 2.3 Hospital Admission and Population Data

We obtained annual county‐level respiratory, cardiovascular, and asthma hospital admission rates for 2017 across California from the U.S. EPA's Benefits Mapping and Analysis Program‐Community Edition, which calculates hospitalization rates, per 100,000 population, using data from the Healthcare and Cost Utilization Project (Sacks et al., 2018). We then converted these annual rates into daily rates by dividing by 365 (Text S2 and Figure S3). California census tract‐level population data for 2017 were obtained from the U.S. Census Bureau.

### 2.4 Impact Assessment Methods

We calculated the number of excess respiratory, cardiovascular, and asthma hospital admissions attributable to fire‐originated PM_2.5_ in central California on each day between October 8 and 20, 2017. We constrained the study area to central California given the geographic extent of the CMAQ model. October 8–20 was identified as the fire period based on recorded fire activity.

To estimate the total attributable hospitalizations, we used a health impact function that is appropriate when evaluating health impacts attributed to wildfires relative to the long‐term hospital admission rates (Borchers‐Arriagada, Palmer, Bowman, Morgan, et al., 2020; Borchers‐Arriagada, Palmer, Bowman, Williamson, et al., 2020; Bowman et al., 2019; Broome et al., 2016; Fann et al., 2013, 2018; Johnston et al., 2021), rather than relative to the elevated admission rates during the fire period, for which we do not have data. The health impact function, derived from a log linear model for the relationship between the exposure and health outcome

(1) $$\Delta Y(s,\;t)=Y_{NF}(s)\times (e^{\beta \times \Delta X(s,\;t)}-1)\times Pop(s)$$

determines the number of attributable hospital admissions per square kilometer, ΔY(s,t), at location set s on day t, where $\Delta Y\left(s,\;t\right)=\;Y\left(s,\;t\right)-\;Y_{NF}\left(s\right)$. The location set s is taken at a set of points, ${\left(s_{1},\;s_{2},\ldots ,s_{N}\right)}^{T}$ **,** corresponding to the cells on a regular estimation grid. $Y_{NF}\left(s\right)$ is the background daily rate of hospital admissions at location set s (i.e., the admission rate without emissions from the October 2017 wildfires), estimated from the annual rate and interpolated from each county to the estimation grid. Y(s,t) represents the daily rate of hospital admissions including admissions related to pollution from the fires. Pop(s) is the population density in grid cell s, interpolated from each census tract to the estimation grid. The quantity $\left(e^{\beta \;\times \;\Delta X(s,\;t)}-1\right)$ is a positive multiplier, where $e^{\beta \;\times \;\Delta X(s,\;t)}$ is the rate ratio of admissions at concentration ΔX(s,t), which determines the percent increase in admissions from $Y_{NF}\left(s\right)$ due to fire‐originated PM_2.5_ exposure. β is the risk coefficient, where β= $\ln(RR)/10\;\mu {\text{g/m}}^{3}$ and RR is the rate ratio for the CRF, either WF or NF, for a 10 µg/m^3^ increase in 2‐day average PM_2.5_. $\Delta X\left(s,\;t\right)=\;X\left(s,\;t\right)-\;X_{NF}\left(s,\;t\right)$ and is the 2‐day average fire‐originated PM_2.5_ concentration at location set s on day t, where X(s,t) is the 2‐day average total PM_2.5_ concentration, obtained from one of the three exposure surfaces, and $X_{NF}\left(s,\;t\right)$ is the background concentration (i.e., concentrations without emissions from the October 2017 wildfires), either $X_{NF\_2016}\left(s\right)$ or the 2‐day average of $X_{NF\_CMAQ}\left(s,\;t\right)$. A 2‐day average is used for ΔX(s,t) to match the CRFs. The total number of attributable admissions on day t, n(t), is calculated as the sum of excess admissions across all grid cells within central California, $n\left(t\right)=\;\sum_{i}^{N}\Delta Y\left(s_{i},t\right)$.

To identify the most impacted subpopulations using the available CRFs, we stratified the impact assessment results by age for respiratory hospital admissions and by age and sex for asthma hospital admissions. All estimates were rounded to the nearest whole number, instead of two significant figures, given our focus on a small geographic region.

To account for uncertainty in the health impact assessment, we conducted Monte Carlo simulations, using 100,000 iterations, on $X\left(s,t\right)$ and β. For the Monte Carlo simulations, we assumed that the total PM_2.5_ estimations (X(s,t)) and the CRFs (β) follow lognormal distributions. For X(s,t), the uncertainty is defined by the variance of the PM_2.5_ estimate. For β, the uncertainty is defined from the 95% CI of the CRF. We neglect uncertainties in population and background admission rates since they are likely small and not time variant.

### 2.5 Evaluating Sensitivity of Assessment to Inputs

To determine which inputs have the largest impact on the estimated attributable respiratory and cardiovascular hospital admissions and their CIs, we conducted three sensitivity analyses, creating 10 alternative impact assessment approaches which used a different total PM_2.5_ surface, background PM_2.5_ surface, CRF, or sources of uncertainty compared to the base case (Table 1). The base case impact assessment used the following inputs: total exposure estimates from the BME data fusion, CMAQ percent attributable to isolate fire‐originated PM_2.5_, the WF CRF, and uncertainty from both the total PM_2.5_ estimate (X(s,t)) and the CRF (β). This was identified as the base case because we believe it incorporates the most accurate and representative information. For the sensitivity analyses, we only changed one input at a time from the base case to understand the individual impact of each.

**Table 1:** Definition of Inputs for the Three Sensitivity Analyses and 10 Alternative Impact Assessments to Compare to the Base Case

Sensitivity analysis	Total PM_2.5_ estimation	Background PM_2.5_ estimation	CRF type	Sources of uncertainty
Base case	BME data fusion	CMAQ % attributable	WF	CRF and total PM_2.5_
*(1) Sensitivity to fire‐originated PM_2.5_ estimation*	CC‐CMAQ	CMAQ % attributable	WF	CRF and total PM_2.5_
	BME kriging	CMAQ % attributable	WF	CRF and total PM_2.5_
	BME data fusion	October 2016	WF	CRF and total PM_2.5_
*(2) Sensitivity to CRF type*	BME data fusion	CMAQ % attributable	NF	CRF and total PM_2.5_
*(3) Sensitivity to sources of uncertainty*	BME data fusion	CMAQ % attributable	WF	CRF
	BME data fusion	CMAQ % attributable	WF	Total PM_2.5_
	CC‐CMAQ	CMAQ % attributable	WF	CRF
	CC‐CMAQ	CMAQ % attributable	WF	Total PM_2.5_
	BME kriging	CMAQ % attributable	WF	CRF
	BME kriging	CMAQ % attributable	WF	Total PM_2.5_

The first sensitivity analysis evaluated the impact of the wildfire PM_2.5_ exposure estimates on the estimated excess hospital admissions and 95% CIs. For this, we first compared the base case total PM_2.5_ concentration surface (BME data fusion) to two simpler alternatives: CC‐CMAQ and BME kriging of observations. We next compared the base case background PM_2.5_ surface (CMAQ percent attributable) to one alternative: the October 2016 approach. The second sensitivity analysis evaluated the impact of the CRF on the estimated excess hospital admissions and their CIs. For this, we compared the WF CRF used in the base case to the ambient NF CRF. The third sensitivity analysis aimed to identify the primary sources of uncertainty in the hospital admissions estimates. For this, we compared the estimated admissions' confidence bounds when uncertainty from both the total PM_2.5_ concentration surface (X(s,t)) and the CRF (β) was accounted for, to when uncertainty from only X(s,t) or β was accounted for. We conducted this sensitivity analysis for all three total PM_2.5_ surfaces to determine if the primary sources of uncertainty changed depending on the exposure estimate used.

## 3 Results

### 3.1 Impact of Wildfire PM_2.5_ Exposure on Hospital Admissions

Using base case assumptions, we estimate there were an excess 240 (95% CI: 114, 404), 68 (95% CI: −10, 159), and 45 (95% CI: 18, 81) respiratory, cardiovascular, and asthma hospital admissions, respectively, attributable to fire‐originated PM_2.5_ exposure between October 8 and 20 (Table 2). More than half of the total respiratory and asthma hospital admissions were people over the age of 65, who comprise only 14% of the total population but have higher background rates of hospital admissions. Two thirds of the excess asthma admissions were female, who have a higher risk of asthma‐related hospitalization. The least impacted age groups for both respiratory and asthma admissions were people aged 5–19. While asthma hospital admissions have the highest risk coefficient for wildfire PM_2.5_ exposure, they account for the lowest number of attributable admissions due to the low background admission rates. It is important to note that the admission counts for the age and sex subgroups do not sum to the all ages, all sexes count because different CRFs are used for each subgroup and are not designed to total to the number calculated using the general population CRF.

**Table 2:** Number of Excess Respiratory, Cardiovascular, and Asthma Hospital Admissions Attributable to Wildfire‐Originated PM_2.5_, October 8–20, 2017, Estimated Using Base Case Assumptions

	RR (95% CI)^a^	# Admissions (95% CI)
Respiratory hospital admissions		
All ages	1.028 (1.014, 1.041)	240 (114, 404)
Ages 0–4	1.045 (1.010, 1.082)	27 (6, 54)
Ages 5–19	1.027 (0.984, 1.076)	15 (−9, 43)
Ages 20–64	1.024 (1.005, 1.044)	65 (13, 131)
Ages 65–99	1.030 (1.011, 1.049)	126 (44, 232)
Cardiovascular hospital admissions		
All ages	1.008 (0.999, 1.018)	68 (−10, 159)
Asthma hospital admissions		
All ages and sexes	1.048 (1.021, 1.076)	45 (18, 81)
Male	1.031 (0.990, 1.073)	14 (−4, 35)
Female	1.059 (1.022, 1.097)	29 (10, 55)
Ages 0–4	1.083 (1.021, 1.149)	7 (2, 15)
Ages 5–19	0.999 (0.935, 1.068)	0 (−10, 11)
Ages 20–64	1.041 (0.995, 1.090)	17 (−2, 40)
Ages 65–99	1.101 (1.030, 1.178)	27 (7, 57)

^a^ Rate ratio per 10 µg/m^3^ increase in 2‐day average PM_2.5_ (Delfino et al., 2009).

**Figure 1:**
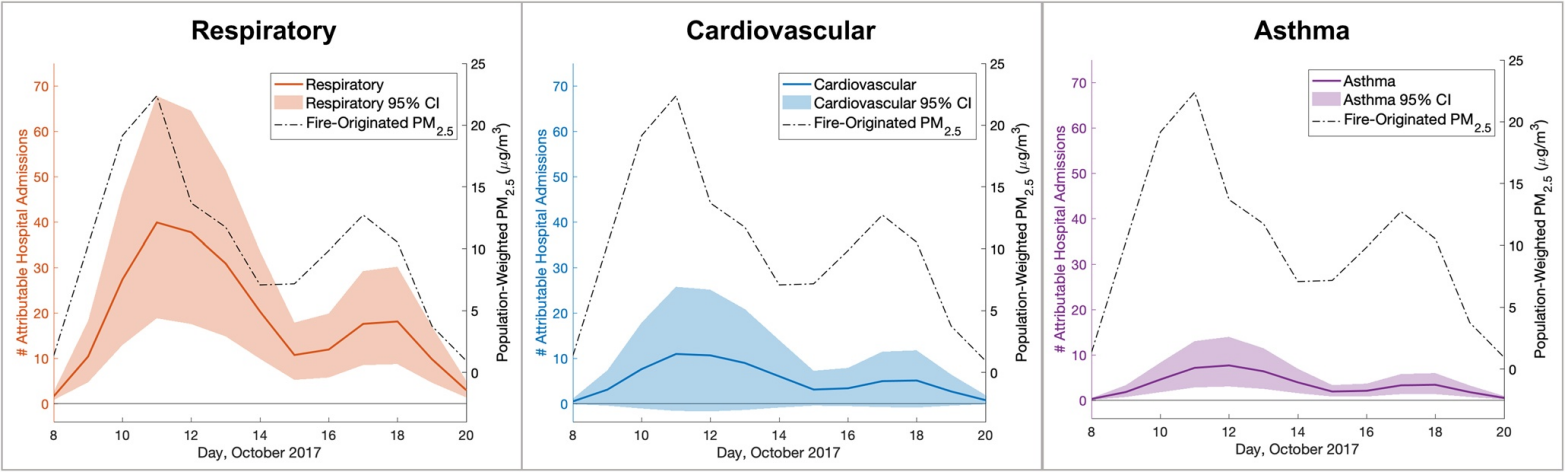
Daily excess respiratory, cardiovascular, and asthma hospital admissions attributable to wildfire PM_2.5_ and daily population‐weighted average fire‐originated PM_2.5_ concentrations, October 8–20, 2017, estimated using base case assumptions.

Hospital admissions varied daily, reflecting variation in smoke exposure (Figure 1). Between October 8 and 20, October 11 had the highest number of excess respiratory and cardiovascular admissions, 40 (95% CI: 19, 68) and 11 (95% CI: −2, 26), respectively. October 12 had the most excess asthma admissions, with 8 (95% CI: 3, 14) total admissions. The days with the highest number of admissions have the largest uncertainty around the estimates. The peaks in excess admissions occurred the same day as or 1 day after peaks in population‐weighted fire‐originated PM_2.5_ concentrations, a reflection of the CRFs which report an increase in attributable admissions per increase in 2‐day average PM_2.5_. There were two peaks in excess admissions, October 11–12 and October 18, reflecting peaks in smoke concentrations. Daily counts of admissions for each health endpoint can be found in supporting information (Text S3 and Table S1).

**Figure 2:**
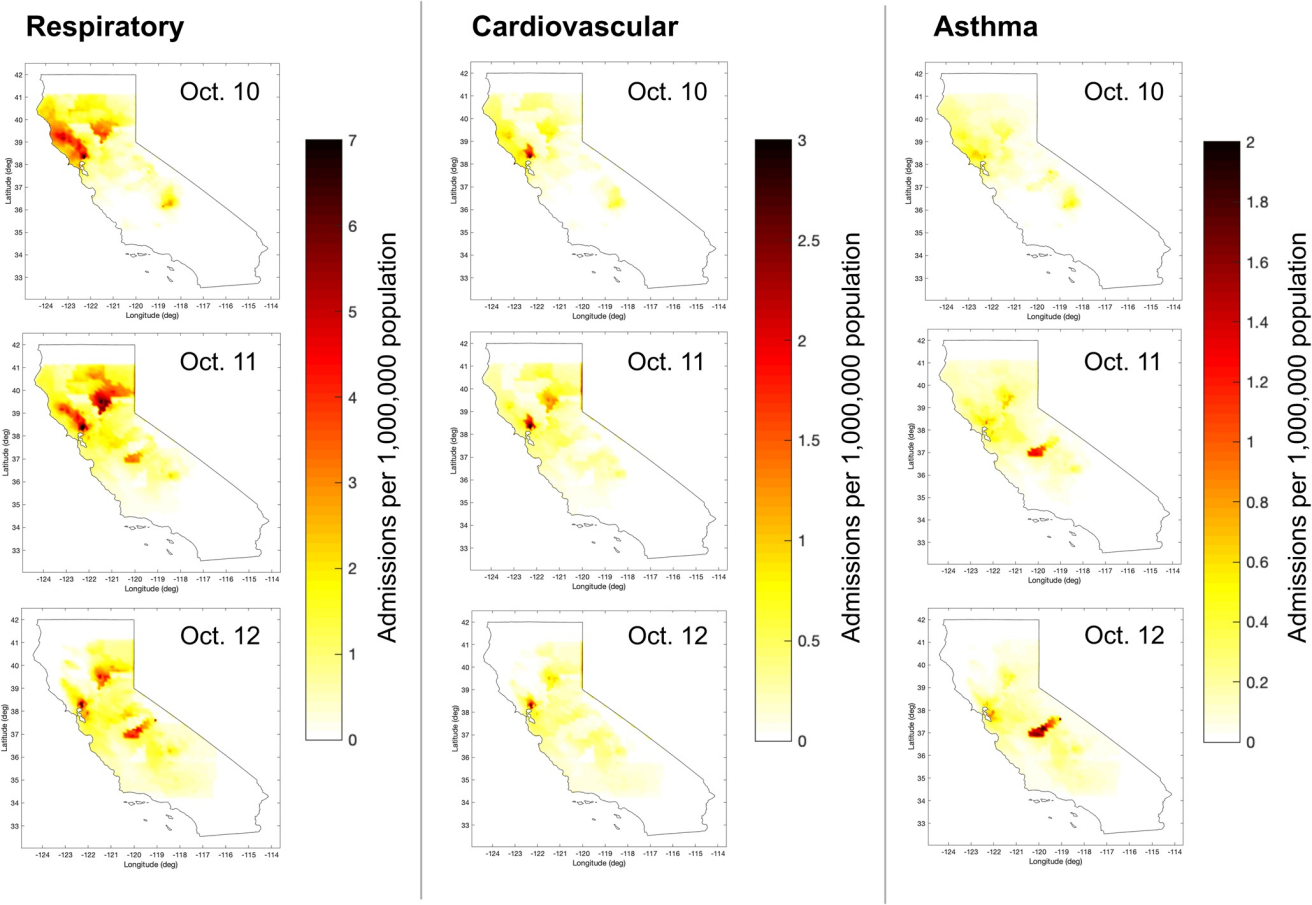
Excess respiratory, cardiovascular, and asthma hospital admissions attributable to wildfire PM_2.5_, expressed as rate per 1,000,000 person‐days, October 10–12, 2017, estimated using base case assumptions.

To understand the spatial distribution of hospital admissions across central California during the fires, we mapped the rate of the excess admissions (Figure 2) and the percent increase in hospital admissions due to wildfire PM_2.5_ exposure (Text S4 and Figure S4) and calculated county‐level attributable admissions (Table 3, Text S5, and Figure S5). Overall, attributable respiratory hospital admissions occurred at a higher rate in comparison to cardiovascular and asthma admissions, aligning with the results in Tables 2 and 3, and were more widespread across multiple counties. Between October 10 and 12, the regions with the highest rates of attributable admissions varied by health outcome. For respiratory hospital admissions, some of the highest rates occurred in Napa, Mendocino, Butte, Madera, and Contra Costa Counties. For cardiovascular and asthma hospital admissions, the highest rates occurred in Napa and Butte Counties and Madera and Alameda Counties, respectively. The counties with the highest number of excess admissions also varied by health outcome, with 56% of all respiratory and cardiovascular admissions occurring in the Bay Area (Alameda, Contra Costa, Marin, Napa, San Francisco, San Mateo, Santa Clara, Solano, and Sonoma Counties). Napa County had the most respiratory (12.9%) and cardiovascular admissions (17.6%), while Alameda County had the most asthma admissions (17.8%).

**Table 3:** Number of Fire‐Attributable Respiratory, Cardiovascular, and Asthma Hospital Admissions, Average Fire‐Originated PM_2.5_ Concentrations, and Total Population in the 10 Counties With the Most Excess Admissions, October 8–20, 2017, Estimated Using Base Case Assumptions

County	# Admissions (95% CI)			Average fire‐originated PM_2.5_ (Std. Dev.) (µg/m^3^)^a^	Total population (# persons)^b^
	Respiratory	Cardiovascular	Asthma		
Napa	31 (15, 48)	12 (−2, 26)	3 (1, 5)	39.57 (17.73)	141,005
Santa Clara	25 (12, 42)	8 (−1, 20)	5 (2, 10)	10.50 (0.85)	1,911,226
Alameda	25 (12, 42)	6 (−1, 14)	8 (3, 14)	11.94 (2.77)	1,629,615
Contra Costa	17 (8, 28)	4 (−1, 10)	4 (2, 8)	14.54 (3.92)	1,123,678
Sacramento	12 (6, 21)	4 (−1, 10)	3 (1, 5)	10.00 (1.03)	1,495,400
Fresno	11 (5, 18)	4 (−1, 9)	2 (1, 4)	9.03 (2.01)	971,616
Solano	11 (5, 19)	3 (0, 7)	2 (1, 4)	19.64 (6.87)	434,981
Sonoma	10 (4, 17)	3 (0, 6)	2 (1, 3)	25.54 (5.21)	500,943
Butte	10 (5, 18)	2 (0, 5)	1 (0, 2)	9.67 (1.56)	225,207
San Joaquin	9 (4, 15)	2 (0, 5)	2 (1, 3)	10.18 (0.46)	724,153

^a^ The population‐weighted average and standard deviation of the 1‐km resolution fire‐originated PM_2.5_ estimations across the county, October 8–20, 2017.

^b^ County‐level total population calculated using 2017 census tract‐level population data.

The counties with high excess admission rates and counts are determined by three primary factors: the fire‐originated PM_2.5_ concentrations, background hospital admission rates, and population density. Since the smoke exposure estimates remain the same for all health outcomes, the differences in where high attributable admission rates are located are driven by the county‐level background admission rates. For example, Napa County was exposed to some of the highest PM_2.5_ concentrations during the 2017 wildfires (Cleland et al., 2020), with more than a 30% increase in respiratory and asthma admissions due to fire‐originated PM_2.5_ exposure (Figure S4). While Napa County also had the highest rates of excess respiratory admissions, it had notably lower rates of excess asthma admissions due to a low background rate of asthma hospital admissions in 2017. Further, the differences in total attributable admissions are driven by population density. For example, while sparsely populated Madera County had the highest rates of excess asthma admissions, densely populated Alameda County, which had comparably lower attributable admission rates, had the most excess asthma admissions.

### 3.2 Sensitivity of Assessment to Inputs

To identify which impact assessment inputs had the most impact on the magnitude and uncertainty of the admissions estimates, we compared four different approaches that used a different total PM_2.5_ estimation method, background PM_2.5_ estimation method, or CRF relative to the base case. Overall, the health impact estimates are sensitive to all three inputs, but the overlapping CIs indicate that the sensitivity is not statistically significant (Table 4). Changes in the magnitude and range of uncertainty of the admissions estimated are present both across the entire fire period (Table 4) and on each day (Text S6 and Figure S6). The locations of the excess admissions also change depending on the impact assessment approach used (Text S6 and Figure S7).

**Table 4:** Comparison of Methods for Estimating Excess Respiratory and Cardiovascular Hospital Admissions and Fire‐Originated PM_2.5_ Exposure, October 8–20, 2017

	Impact assessment method			# Admissions (95% CI)		Fire‐originated PM_2.5_ (µg/m^3^)^a^		
	Total PM_2.5_ estimation	Background PM_2.5_ estimation	CRF type	Respiratory	Cardiovascular	Population‐weighted average (Std. Dev.)	Spatial average (Std. Dev.)	95th percentile
1^b^	BME data fusion	CMAQ % attributable	WF^c^	240 (114, 404)	68 (−10, 159)	10.05 (6.58)	7.05 (9.81)	26.59
2	CC‐CMAQ	CMAQ % attributable	WF	251 (77, 620)	70 (−10, 211)	9.84 (6.10)	6.56 (9.63)	27.47
3	BME kriging	CMAQ % attributable	WF	280 (124, 512)	78 (−12, 192)	11.02 (7.08)	8.19 (11.17)	26.40
4	BME data fusion	October 2016	WF	299 (126, 544)	84 (−13, 208)	8.77 (7.50)	6.56 (8.48)	22.65
5	BME data fusion	CMAQ % attributable	NF^d^	177 (87, 305)	163 (95, 261)	10.05 (6.58)	7.05 (9.81)	26.59

^a^ The population‐weighted average and standard deviation, spatial average and standard deviation, and 95th percentile of the 1‐km resolution fire‐originated PM_2.5_ estimations across central California.

^b^ Base case impact assessment.

^c^ Wildfire‐specific CRFs (rate ratio per 10 μg/m^3^ increase in 2‐day average PM_2.5_)—respiratory: 1.028 (95% CI: 1.014, 1.041); cardiovascular: 1.008 (95% CI: 0.999, 1.018) (Delfino et al., 2009).

^d^ Nonwildfire‐specific CRFs (rate ratio per 10 μg/m^3^ increase in 2‐day average PM_2.5_)—respiratory: 1.021 (95% CI 1.012, 1.030); cardiovascular: 1.019 (95% CI: 1.013, 1.025) (Zanobetti et al., 2009).

Of the three inputs, the NF CRFs have the largest impact on the magnitude of the estimate compared to the base case, with a 26% decrease and 140% increase in the estimated number of excess respiratory and cardiovascular admissions, respectively. These changes are due to the NF CRFs having a lower risk coefficient for respiratory and considerably higher risk coefficient for cardiovascular hospital admissions compared to the WF CRFs. The widths of confidence bounds shrink slightly when using the NF CRFs, due to their narrower 95% CIs. The CRFs' large impact on the estimated excess admissions emphasizes the importance of using a WF CRF, especially as the PM_2.5_ composition of smoke affects toxicity (Liu & Peng, 2019).

The method used to estimate background PM_2.5_ also impacts the magnitude and uncertainty of admissions estimates. When the October 2016 approach is used to isolate fire‐originated PM_2.5_ instead of the CMAQ percent attributable approach, there is a 25% and 24% increase in the excess respiratory and cardiovascular admissions estimated, respectively, and a 44% and 31% increase in the CI width, respectively. Using the October 2016 surface increases the frequency of low estimated fire‐originated PM_2.5_ concentrations, shown by the reduction in both the spatial and population‐weighted average and the 95th percentile of concentrations. This shift in the distribution of smoke concentrations across California during the fires increases the number and associated uncertainty of excess admissions estimated.

Changing the method for estimating total exposure to either CC‐CMAQ or BME kriging has the least impact on magnitude but the largest impact on uncertainty, especially for respiratory admissions. CC‐CMAQ increases the estimated excess respiratory and cardiovascular admissions by 5% and 3%, respectively, and the width of the CIs by 87% and 31%, respectively. There is a slight increase in admissions because, compared to BME data fusion, CC‐CMAQ has a higher frequency of high estimated smoke concentrations, shown by the increase in the 95th percentile of fire‐originated PM_2.5_. BME kriging increases the estimated excess respiratory and cardiovascular admissions by 16% and 15%, respectively, and increases the width of the CIs by 33% and 21%, respectively. This increase in admissions occurs because, compared to BME data fusion, BME kriging on average estimates higher fire‐originated PM_2.5_ concentrations across central California. The observed decrease in the impact assessment results' precision when either BME kriging or CC‐CMAQ is used occurs because both exposure surfaces have higher estimation variance than BME data fusion (Cleland et al., 2020).

**Figure 3:**
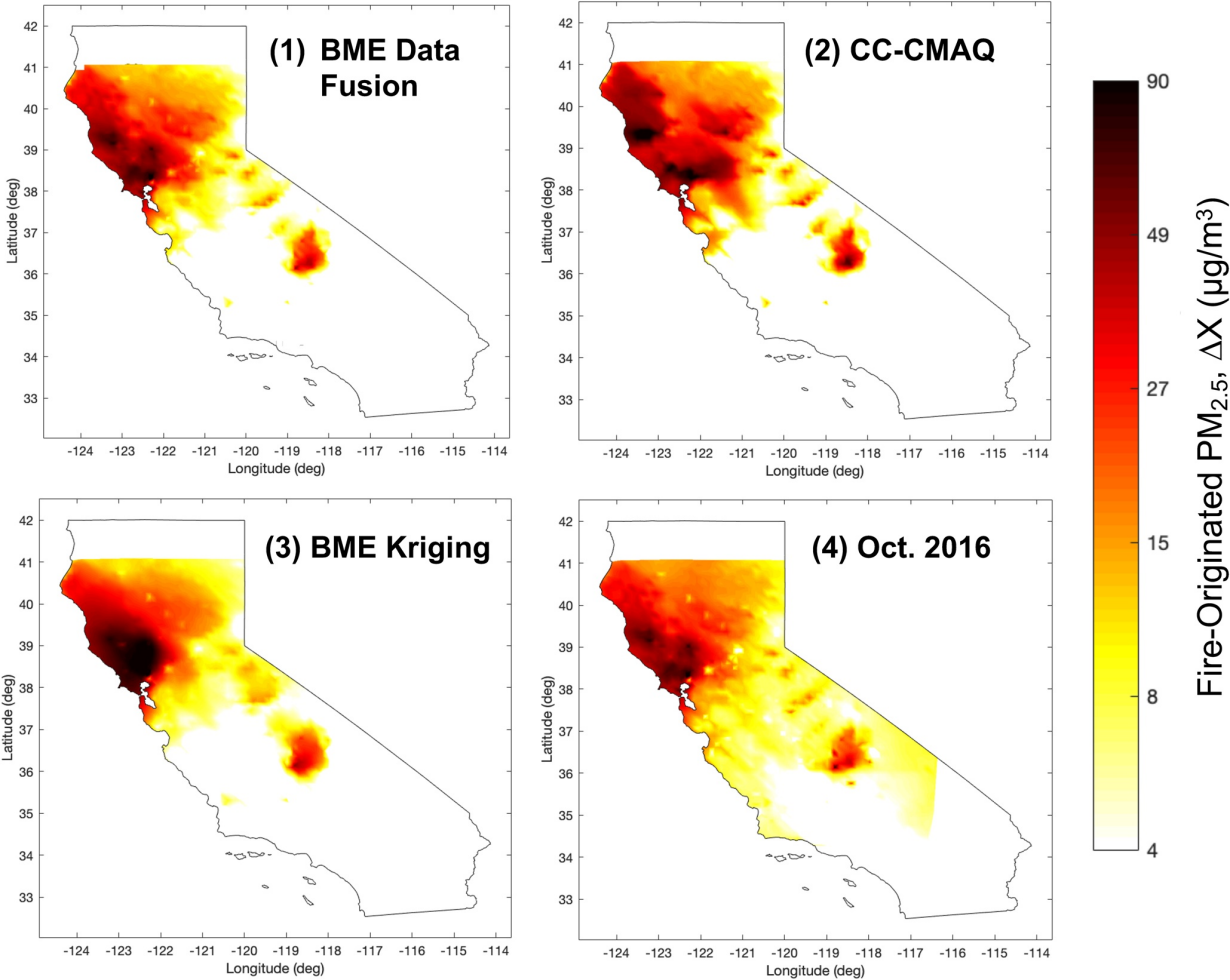
Comparison of methods for estimating exposure to fire‐originated PM_2.5_ on October 10, 2017. (1) Base case, BME data fusion with CMAQ % attributable as background; (2) CC‐CMAQ with CMAQ % attributable as background; (3) BME kriging with CMAQ % attributable as background; and (4) BME data fusion with October 2016 as background. BME, Bayesian Maximum Entropy; CMAQ, Community Multiscale Air Quality; CC‐CMAQ, corrected Community Multiscale Air Quality model output, using the Constant Air Quality Model Performance.

To further understand how estimates of fire‐originated PM_2.5_ exposure differed between methods, we compared the four exposure surfaces spatially on October 10 (Figure 3) and temporally between October 8 and 20 (Text S6 and Figure S8). Using BME kriging or CC‐CMAQ to estimate total exposure instead of BME data fusion changes the smoke plume characteristics, impacting both the location and magnitude of concentrations and the number of attributable admissions estimated. Both CC‐CMAQ and BME kriging have larger plumes of high fire‐originated concentrations north of San Francisco Bay compared to BME data fusion, partly explaining the increase in estimated admissions. When the October 2016 approach is used instead of the CMAQ percent attributable approach to estimate background concentrations, there appears to be less clear isolation of the smoke plumes, with widespread low fire‐originated PM_2.5_ concentrations south and east of the Bay Area. The increased area covered by wildfire PM_2.5_ using this method partly explains the increase in estimated admissions. It is possible that ambient, nonfire PM_2.5_ across California changed between 2016 and 2017, which would limit the October 2016 surface's ability to accurately isolate smoke concentrations.

**Figure 4:**
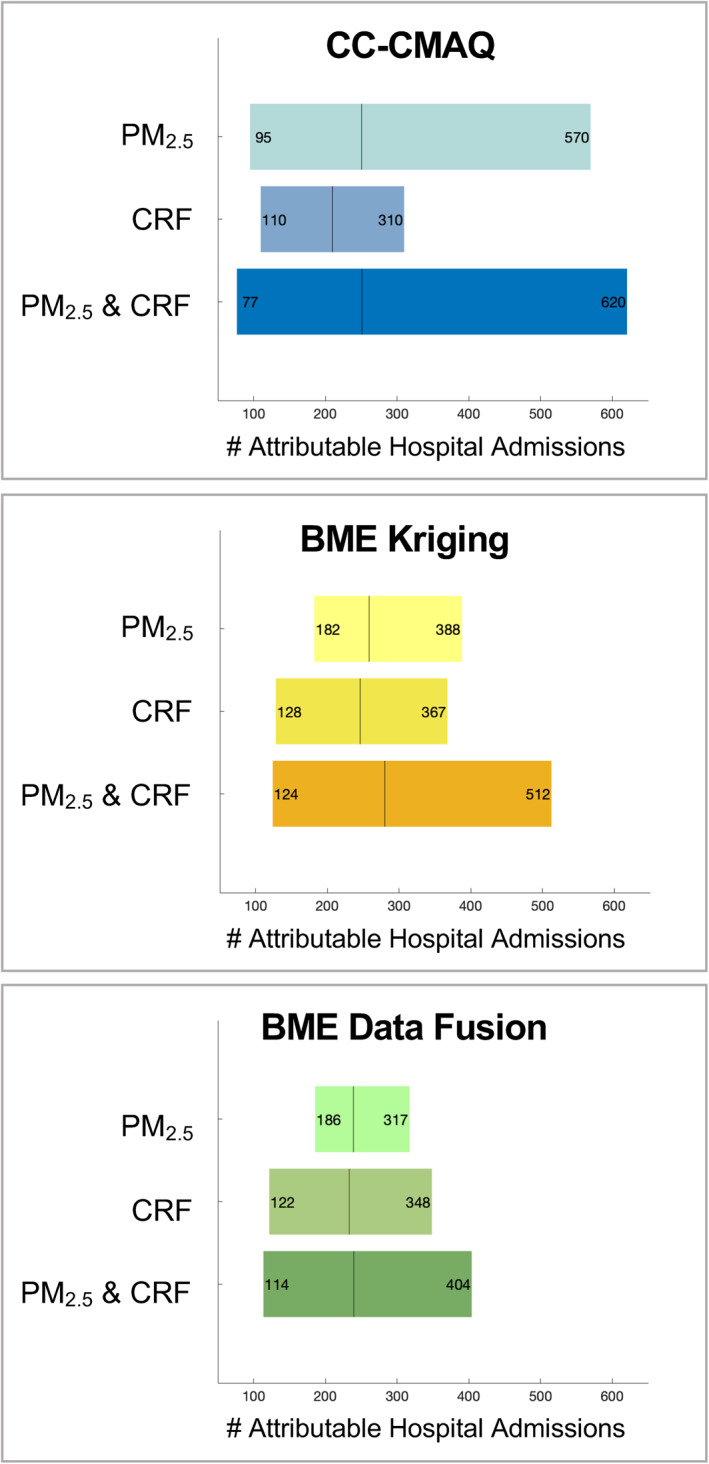
The individual contributions of uncertainties in the CRF and total PM_2.5_ surface to the total uncertainty in estimated respiratory hospital admissions when uncertainties in both the CRF and total PM_2.5_ surface are accounted for. Uncertainties are shown as 95% confidence intervals with the vertical line marking the mean estimate. Results are shown using the three total PM_2.5_ exposure estimates (CC‐CMAQ, BME kriging, and BME data fusion), which all use CMAQ % attributable for the background concentrations and the WF CRF. CRF, concentration–response function; CC‐CMAQ, Constant Air Quality Model Performance‐corrected Community Multiscale Air Quality model output; BME, Bayesian Maximum Entropy; WF, wildfire specific.

We then quantified, for each of the three total exposure surfaces, the contributions of uncertainties in the total PM_2.5_ estimate and CRF to the overall uncertainty of the number of excess respiratory admissions estimated (Figure 4). Only accounting for one source of uncertainty, regardless of the exposure surface used, produces optimistically small confidence bounds and underestimates the admissions estimates' uncertainty. When uncertainty from both the total exposure estimate and CRF is accounted for, CC‐CMAQ, the least precise PM_2.5_ estimate, produces the least precise admissions estimates. BME data fusion, the most precise PM_2.5_ estimate, produces the most precise admissions estimates. With BME data fusion, the majority of uncertainty in the admissions estimate comes from the CRF; accounting for the CRF's uncertainty, when uncertainty from the PM_2.5_ exposure surface is already accounted for, increases the CI width by 121%. In comparison, the primary source of uncertainty when using CC‐CMAQ is the exposure surface; accounting for the CRF's uncertainty, when uncertainty from the PM_2.5_ exposure surface is already accounted for, only increases the CI width by 14%. The exposure surface and the CRF contribute comparably to uncertainty when BME kriging is used. In health impact assessments, when using CRFs from previously published literature, the uncertainty of the CRF is predefined. In this case, when there is not control over the CRF's uncertainty, there is often control over the uncertainty of the exposure surface, especially if it is developed for the purpose of the assessment. Reducing the variance of the exposure estimate, which BME data fusion does by incorporating three different data sets into the PM_2.5_ estimation surface, not only changes the primary source of uncertainty but also increases the overall precision of the final respiratory admissions estimate. Similarly, when estimating excess cardiovascular hospital admissions using BME data fusion, the CRF contributes the vast majority of uncertainty to the final admissions estimate (Text S6 and Figure S9).

## 4 Discussion and Conclusions

Using the base case impact assessment, we estimate that exposure to fire‐originated PM_2.5_ during the October 2017 wildfires accounted for over 300 excess respiratory and cardiovascular hospital admissions, with the majority of admissions occurring in the Bay Area. The regions with the highest estimated rates and number of attributable admissions were those with extreme smoke concentrations and high background admission rates and population density, such as Napa and Alameda County, where 14% and 10% of the total estimated respiratory and cardiovascular admissions were associated, respectively. While there is only an estimated 1%–4% increase in respiratory and cardiovascular hospital admissions per 10 µg/m^3^ increase in PM_2.5_, since wildfires elevate PM_2.5_ concentrations to hazardous levels across large geographic regions for multiple weeks, the overall health impact of fire events can be noticeable and widespread.

We also show that choices of inputs used in the health impact assessment have an impact on both the magnitude and uncertainty of the number of attributable admissions estimated. In our assessment, the CRF had the greatest impact on the number of excess admissions estimated, while the uncertainty was most impacted by the exposure surface. Regardless of the health effect being studied, the impact of the CRF will depend on which CRFs are selected. Our findings indicate that using a NF CRF may underrepresent respiratory admissions and overrepresent cardiovascular admissions attributable to fire‐originated PM_2.5_. Since there is often variety between CRFs for ambient versus wildfire PM_2.5_ exposure, we recommend that future impact assessments of smoke exposure use a WF CRF when available.

The method used to isolate fire‐originated PM_2.5_ also has implications when assessing the acute health impacts of smoke exposure. Since ambient PM_2.5_ and other meteorological conditions in the same geographic region can change over time, using a CTM run during the fire period without fire emissions, rather monitoring station observations in the study area during a nonfire period, may be able to more realistically isolate fire‐originated PM_2.5_. Additionally, while prior acute health impacts assessments of wildfires have primarily used either monitoring station observations or CTM output to estimate total exposure, we show that combining multiple PM_2.5_ data sets through data fusion reduces the variance of the exposure estimate and increases the precision of the excess admissions estimates. Using multiple PM_2.5_ data sets also often increases the accuracy of the exposure surface (Cleland et al., 2020; Lassman et al., 2017; Reid et al., 2015), in turn likely increasing the accuracy of the estimated acute health impacts.

We further show that accounting for uncertainty from only the CRF or the exposure estimate leads to an underestimation of the health impact estimates' uncertainty, emphasizing the importance of accounting for multiple sources of uncertainty. In many prior assessments that only propagated uncertainty using the CRF's distribution (Borchers‐Arriagada, Palmer, Bowman, Morgan, et al., 2020; Borchers‐Arriagada, Palmer, Bowman, Williamson, et al., 2020; Broome et al., 2016; Fann et al., 2013, 2018; Matz et al., 2020), the confidence bounds around the admissions estimates may be optimistically small. Since PM_2.5_ exposure surfaces are inherently uncertain, it is important to include them as a source of uncertainty to obtain more realistic CIs.

Once uncertainties from both are accounted for, the importance of a good exposure surface becomes more evident. A less precise estimate of total PM_2.5_, like CC‐CMAQ, contributes far more uncertainty to the final health impact estimates than a more precise exposure estimate, like the BME data fusion output. A prior health impact assessment similarly found that when using a calibrated CMAQ model to estimate WF PM_2.5_ exposure, the PM_2.5_ surface contributed more uncertainty than the CRF to the final health impact estimate (Jiang & Yoo, 2019). Since CRFs and their parameterized uncertainty are often drawn from published epidemiological studies, one simple way to reduce the overall uncertainty of health impact assessments is to improve the quality of the exposure surfaces used. To further improve impact assessments and obtain a clearer understanding of the health impacts of wildfires, we recommend that epidemiological studies also use more accurate and informed exposure estimates that integrate multiple PM_2.5_ data sets to increase the accuracy and precision of CRFs. In order to improve access to more advanced smoke exposure estimates, it is necessary to increase collaboration and data sharing among those investigating the air quality and health impacts of fires.

While our study has many strengths, there are also some important limitations. First, we are unable to validate the accuracy of our attributable admissions estimates because we do not know the number of daily hospital admissions that occurred during October 2017, nor do we know how many were attributable to smoke exposure. Further, our analyses only focused on the central California region, given the bounds of the CMAQ model. By limiting the geographical bounds of our analysis, we likely underestimate the total number of admissions attributable to the October 2017 wildfires, since smoke from the fires impacted regions beyond central California. We also do not account for locations that were evacuated due to the fires, nor for time‐activity patterns among the exposed population, and as a result may have under or overestimated population exposure and the number of excess admissions. Additionally, the exposure, background admission rate, and population data all have different geographic scales which may introduce additional uncertainty into our analyses. Finally, we do not account for uncertainty from the estimation of background concentrations.

By showing that health impact estimates are sensitive to different exposure and epidemiological inputs, and by demonstrating the importance of accounting for multiple sources of uncertainty, employing context‐specific CRFs, and using more advanced PM_2.5_ exposure estimates, our work can help improve the quality of health impact assessments, for wildfire smoke and for other exposures, moving forward. Using these insights in combination with available resources, future impact assessments can better identify the appropriate data and methods for estimating, with greater certainty, how extreme air pollution events impact hospitals, EDs, and the population's health. These more informed and realistic estimates of the health impacts of wildfires can better inform decision‐making processes and improve public health evaluation before, during, and in the aftermath of fire events.

## Conflict of Interest

The authors declare no conflicts of interest relevant to this study.

## Supporting information

Supporting Information S1

## Data Availability

The PM_2.5_ data sets used in this research are available at https://doi.org/10.15139/S3/1SRKDN.

## References

[gh2253-bib-0001] Bay Area Air Quality Management District . (2019). Annual Bay Area air quality summaries. Retrieved from https://www.baaqmd.gov/about-air-quality/air-quality-summaries (January 23, 2020).

[gh2253-bib-0002] Boegelsack, N. , Withey, J. , O'Sullivan, G. , & McMartin, D. (2018). A critical examination of the relationship between wildfires and climate change with consideration of the human impact. Journal of Environmental Protection, 9(5), 461–467. 10.4236/jep.2018.95028

[gh2253-bib-0003] Borchers‐Arriagada, N. , Horsley, J. A. , Palmer, A. J. , Morgan, G. G. , Tham, R. , & Johnston, F. H. (2019). Association between fire smoke fine particulate matter and asthma‐related outcomes: Systematic review and meta‐analysis. Environmental Research, 179, 108777. 10.1016/j.envres.2019.108777 31593836

[gh2253-bib-0005] Borchers‐Arriagada, N. , Palmer, A. J. , Bowman, D. M. J. S. , Williamson, G. J. , & Johnston, F. H. (2020). Health impacts of ambient biomass smoke in Tasmania, Australia. International Journal of Environmental Research and Public Health, 17(9), 3264. 10.3390/ijerph17093264 PMC7246513 32392847

[gh2253-bib-0004] Borchers‐Arriagada, N. , Palmer, A. J. , Bowman, D. M. , Morgan, G. G. , Jalaludin, B. B. , & Johnston, F. H. (2020). Unprecedented smoke‐related health burden associated with the 2019–20 bushfires in eastern Australia. Medical Journal of Australia, 213(6), 282–283. 10.5694/mja2.50545 32162689

[gh2253-bib-0006] Bowman, D. M. J. S. , Moreira‐Muñoz, A. , Kolden, C. A. , Chávez, R. O. , Muñoz, A. A. , Salinas, F. , et al. (2019). Human–environmental drivers and impacts of the globally extreme 2017 Chilean fires. Ambio, 48(4), 350–362. 10.1007/s13280-018-1084-1 30128860 PMC6411810

[gh2253-bib-0007] Broome, R. A. , Johnston, F. H. , Horsley, J. , & Morgan, G. G. (2016). A rapid assessment of the impact of hazard reduction burning around Sydney, May 2016. Medical Journal of Australia, 205(9), 407–408. 10.5694/mja16.00895 27809737

[gh2253-bib-0008] Christakos, G. (1990). A Bayesian/maximum‐entropy view to the spatial estimation problem. Mathematical Geology, 22(7), 763–777. 10.1007/BF00890661

[gh2253-bib-0009] Christakos, G. , Bogaert, P. , & Serre, M. L. (2002). Temporal GIS: Advanced functions for field‐based applications. New York, NY: Springer‐Verlag.

[gh2253-bib-0010] Cleland, S. E. , West, J. J. , Jia, Y. , Reid, S. , Raffuse, S. , O'Neill, S. , & Serre, M. L. (2020). Estimating wildfire smoke concentrations during the October 2017 California fires through BME space/time data fusion of observed, modeled, and satellite‐derived PM_2.5_ . Environmental Science and Technology, 54(21), 13439–13447. 10.1021/acs.est.0c03761 33064454 PMC7894965

[gh2253-bib-0011] Deflorio‐Barker, S. , Crooks, J. , Reyes, J. , & Rappold, A. G. (2019). Cardiopulmonary effects of fine particulate matter exposure among older adults, during wildfire and non‐wildfire periods, in the United States 2008–2010. Environmental Health Perspectives, 127(3), 37006. 10.1289/EHP3860 30875246 PMC6768318

[gh2253-bib-0012] Delfino, R. J. , Brummel, S. , Wu, J. , Stern, H. , Ostro, B. , Lipsett, M. , et al. (2009). The relationship of respiratory and cardiovascular hospital admissions to the southern California wildfires of 2003. Occupational and Environmental Medicine, 66(3), 189–197. 10.1136/oem.2008.041376 19017694 PMC4176821

[gh2253-bib-0013] de Nazelle, A. , Arunachalam, S. , & Serre, M. L. (2010). Bayesian maximum entropy integration of ozone observations and model predictions: An application for attainment demonstration in North Carolina. Environmental Science & Technology, 44(15), 5707–5713. 10.1021/es100228w 20590110 PMC2912419

[gh2253-bib-0014] Fann, N. , Alman, B. , Broome, R. A. , Morgan, G. G. , Johnston, F. H. , Pouliot, G. , & Rappold, A. G. (2018). The health impacts and economic value of wildland fire episodes in the U.S. 2008–2012. Science of the Total Environment, 610–611, 802–809. 10.1016/j.scitotenv.2017.08.024 PMC6117838 28826118

[gh2253-bib-0015] Fann, N. , Fulcher, C. M. , & Baker, K. (2013). The recent and future health burden of air pollution apportioned across U.S. sectors. Environmental Science and Technology, 47(8), 3580–3589. 10.1021/es304831q 23506413

[gh2253-bib-0016] Ford, B. , Val Martin, M. , Zelasky, S. E. , Fischer, E. V. , Anenberg, S. C. , Heald, C. L. , & Pierce, J. R. (2018). Future fire impacts on smoke concentrations, visibility, and health in the contiguous United States. GeoHealth, 2, 229–247. 10.1029/2018GH000144 32159016 PMC7038896

[gh2253-bib-0017] Gan, R. W. , Liu, J. , Ford, B. , O'Dell, K. , Vaidyanathan, A. , Wilson, A. , et al. (2020). The association between wildfire smoke exposure and asthma‐specific medical care utilization in Oregon during the 2013 wildfire season. Journal of Exposure Science and Environmental Epidemiology, 30(4), 618–628. 10.1038/s41370-020-0210-x 32051501 PMC8745685

[gh2253-bib-0018] Haikerwal, A. , Akram, M. , Monaco, A. D. , Smith, K. , Sim, M. R. , Meyer, M. , et al. (2015). Impact of fine particulate matter (PM_2.5_) exposure during wildfires on cardiovascular health outcomes. Journal of the American Heart Association, 4(7), e001653. 10.1161/JAHA.114.001653 26178402 PMC4608063

[gh2253-bib-0019] Jaffe, D. A. , O'Neill, S. M. , Larkin, N. K. , Holder, A. L. , Peterson, D. L. , Halofsky, J. E. , & Rappold, A. G. (2020). Wildfire and prescribed burning impacts on air quality in the United States. Journal of the Air and Waste Management Association, 70(6), 583–615. 10.1080/10962247.2020.1749731 32240055 PMC7932990

[gh2253-bib-0020] Jiang, X. , & Yoo, E. H. (2019). Modeling wildland fire‐specific PM_2.5_ concentrations for uncertainty‐aware health impact assessments. Environmental Science and Technology, 53(20), 11828–11839. 10.1021/acs.est.9b02660 31533425

[gh2253-bib-0021] Johnston, F. H. , Borchers‐Arriagada, N. , Morgan, G. G. , Jalaludin, B. , Palmer, A. J. , Williamson, G. J. , & Bowman, D. M. J. S. (2021). Unprecedented health costs of smoke‐related PM_2.5_ from the 2019–20 Australian megafires. Nature Sustainability, 4, 42–47. 10.1038/s41893-020-00610-5

[gh2253-bib-0022] Lassman, W. , Ford, B. , Gan, R. W. , Pfister, G. , Magzamen, S. , Fischer, E. V. , & Pierce, J. R. (2017). Spatial and temporal estimates of population exposure to wildfire smoke during the Washington state 2012 wildfire season using blended model, satellite, and in situ data. GeoHealth, 1, 106–121. 10.1002/2017GH000049 32158985 PMC7007107

[gh2253-bib-0023] Liu, J. C. , Mickley, L. J. , Sulprizio, M. P. , Dominici, F. , Yue, X. , Ebisu, K. , et al. (2016). Particulate air pollution from wildfires in the Western US under climate change. Climatic Change, 138(3–4), 655–666. 10.1007/s10584-016-1762-6 28642628 PMC5476308

[gh2253-bib-0024] Liu, J. C. , & Peng, R. D. (2019). The impact of wildfire smoke on compositions of fine particulate matter by ecoregion in the Western US. Journal of Exposure Science and Environmental Epidemiology, 29(6), 765–776. 10.1038/s41370-018-0064-7 30185941

[gh2253-bib-0025] Liu, J. C. , Pereira, G. , Uhl, S. A. , Bravo, M. A. , & Bell, M. L. (2015). A systematic review of the physical health impacts from non‐occupational exposure to wildfire smoke. Environmental Research, 136, 120–132. 10.1016/j.envres.2014.10.015 25460628 PMC4262561

[gh2253-bib-0026] Liu, J. C. , Wilson, A. , Mickley, L. J. , Dominici, F. , Ebisu, K. , Wang, Y. , et al. (2017). Wildfire‐specific fine particulate matter and risk of hospital admissions in urban and rural counties. Epidemiology, 28(1), 77–85. 10.1097/EDE.0000000000000556 27648592 PMC5130603

[gh2253-bib-0027] Matz, C. J. , Egyed, M. , Xi, G. , Racine, J. , Pavlovic, R. , Rittmaster, R. , et al. (2020). Health impact analysis of PM_2.5_ from wildfire smoke in Canada (2013–2015, 2017–2018). The Science of the Total Environment, 725, 138506. 10.1016/j.scitotenv.2020.138506 32302851

[gh2253-bib-0028] Rappold, A. G. , Cascio, W. E. , Kilaru, V. J. , Stone, S. L. , Neas, L. M. , Devlin, R. B. , & Diaz‐Sanchez, D. (2012). Cardio‐respiratory outcomes associated with exposure to wildfire smoke are modified by measures of community health. Environmental Health, 11(1), 71. 10.1186/1476-069X-11-71 23006928 PMC3506568

[gh2253-bib-0029] Reid, C. E. , Brauer, M. , Johnston, F. H. , Jerrett, M. , Balmes, J. R. , & Elliott, C. T. (2016). Critical review of health impacts of wildfire smoke exposure. Environmental Health Perspectives, 124(9), 1334–1343. 10.1289/ehp.1409277 27082891 PMC5010409

[gh2253-bib-0030] Reid, C. E. , Jerrett, M. , Petersen, M. L. , Pfister, G. G. , Morefield, P. E. , Tager, I. B. , et al. (2015). Spatiotemporal prediction of fine particulate matter during the 2008 northern California wildfires using machine learning. Environmental Science and Technology, 49(6), 3887–3896. 10.1021/es505846r 25648639

[gh2253-bib-0031] Reisen, F. , Duran, S. M. , Flannigan, M. , Elliott, C. , & Rideout, K. (2015). Wildfire smoke and public health risk. International Journal of Wildland Fire, 24(8), 1029–1044. 10.1071/WF15034

[gh2253-bib-0032] Reyes, J. M. , Xu, Y. , Vizuete, W. , & Serre, M. L. (2017). Regionalized PM_2.5_ Community Multiscale Air Quality model performance evaluation across a continuous spatiotemporal domain. Atmospheric Environment, 148, 258–265. 10.1016/J.ATMOSENV.2016.10.048 28848374 PMC5571875

[gh2253-bib-0033] Rittmaster, R. , Adamowicz, W. L. , Amiro, B. , & Pelletier, R. T. (2006). Economic analysis of health effects from forest fires. Canadian Journal of Forest Research, 36(4), 868–877. 10.1139/X05-293

[gh2253-bib-0034] Sacks, J. D. , Lloyd, J. M. , Zhu, Y. , Anderton, J. , Jang, C. J. , Hubbell, B. , & Fann, N. (2018). The Environmental Benefits Mapping and Analysis Program‐Community Edition (BenMAP‐CE): A tool to estimate the health and economic benefits of reducing air pollution. Environmental Modelling & Software, 104, 118–129. 10.1016/j.envsoft.2018.02.009 29962895 PMC6022291

[gh2253-bib-0035] Serre, M. L. , & Christakos, G. (1999). Modern geostatistics: Computational BME analysis in the light of uncertain physical knowledge—The Equus Beds study. Stochastic Environmental Research and Risk Assessment, 13(1–2), 1–26. 10.1007/s004770050029

[gh2253-bib-0036] Spracklen, D. V. , Mickley, L. J. , Logan, J. A. , Hudman, R. C. , Yevich, R. , Flannigan, M. D. , & Westerling, A. L. (2009). Impacts of climate change from 2000 to 2050 on wildfire activity and carbonaceous aerosol concentrations in the western United States. Journal of Geophysical Research, 114, D20301. 10.1029/2008JD010966

[gh2253-bib-0037] Yue, X. , Mickley, L. J. , Logan, J. A. , & Kaplan, J. O. (2013). Ensemble projections of wildfire activity and carbonaceous aerosol concentrations over the western United States in the mid‐21st century. Atmospheric Environment, 77, 767–780. 10.1016/j.atmosenv.2013.06.003 24015109 PMC3763857

[gh2253-bib-0038] Zanobetti, A. , Franklin, M. , Koutrakis, P. , & Schwartz, J. (2009). Fine particulate air pollution and its components in association with cause‐specific emergency admissions. Environmental Health, 8(1), 1–12. 10.1186/1476-069X-8-58 20025755 PMC2807856

