# Supplementary material for: Estimating the Acute Health Impacts of Fire‐Originated PM2.5 Exposure During the 2017 California Wildfires: Sensitivity to Choices of Inputs
Source: Geohealth. 2021 Jul 1;5(7):e2021GH000414. doi: 10.1029/2021GH000414 (PMC8247531; doi:10.1029/2021GH000414)
Supplement: Supplementary file 1 — Supporting Information S1 [file GH2-5-e2021GH000414-s001.pdf]

GeoHealth

Supporting Information for

Estimating the Acute Health Impacts of Fire-Originated $PM_{2.5}$ Exposure during the 2017 California Wildfires: Sensitivity to Choices of Inputs

Stephanie E. Cleland[1,2], Marc L. Serre[1], Ana G. Rappold[3], J. Jason West[1]

[1]Department of Environmental Sciences and Engineering, Gillings School of Global Public Health, University of North Carolina at Chapel Hill, North Carolina, USA. [2]Oak Ridge Institute for Science and Education at the Center for Public Health and Environmental Assessment, Office of Research and Development, United States Environmental Protection Agency, Research Triangle Park, North Carolina, USA. [3]Center for Public Health and Environmental Assessment, Office of Research and Development, United States Environmental Protection Agency, Research Triangle Park, North Carolina, USA.

Contents of this file

Text S1 to S6
Figures S1 to S9
Tables S1 to S2

Text S1.

Figure S1 shows the two background concentration estimates used in the health impact assessment analyses. To use the October 2016 observations to isolate fire-originated $PM_{2.5}$, we downloaded hourly $PM_{2.5}$ observations from 112 FRM/FEM monitoring stations between 1-31 October 2016. We averaged the $PM_{2.5}$ observations across the entire month at each station location and used the BME framework to spatially interpolate the averaged observations and estimate concentrations at a 1-km resolution. Using this approach, the background concentration surface is the same for all days during the fire period. To use the CMAQ percent attributable approach to isolate fire-originated $PM_{2.5}$, we first calculated the percent of $PM_{2.5}$ attributable to background emissions on each day, 8-20 October, using the CMAQ output with and without fire emissions. Figure S2 shows the estimated percent of $PM_{2.5}$ concentrations attributable to fire emissions on 10 October. We then combined the percent attributable estimate with the total exposure estimate to estimate background concentrations. Using this approach, the background concentration estimate is different for each day during the fire period. As shown in Figure S1, the two methods produce dissimilar background concentration surfaces, explaining the differences in each method's ability to isolate the fire-originated $PM_{2.5}$.

Text S2.

Figure S3 shows the background daily rate of respiratory, cardiovascular, and asthma hospital admissions for each county in California in 2017. No county has high background admission rates for all three health outcomes, explaining some of the observed differences in the locations of high excess admissions rates during the fires.

Text S3.

Table S1 shows the estimated number of excess hospital admissions, using base case assumptions, for all three health outcomes on each day during the fires. As discussed in the Results section, 10 October had the highest number of excess respiratory and cardiovascular admissions and 11 October had the highest number of excess asthma admissions. Daily estimates of excess admissions by age and sex subgroup and for the sensitivity analyses are available from the corresponding author upon reasonable request.

Text S4.

Figure S4 shows the fire-attributable percent increase in hospital admissions for each health outcome across central California between 10-12 October, estimated using base case assumptions. In the health impact assessment equation, the percent increase in admissions is determined by the positive multiplier, $(e^{\beta * \Delta X(s,t)} - 1)$. Asthma hospital admissions saw the largest spike during the fires, with a greater than 33% increase in admissions in the Bay Area due to wildfire $PM_{2.5}$ exposure. There was also a rise in respiratory hospital admissions, with a 10-33% increase in admissions in the Bay Area. In comparison, there was only a slight increase in cardiovascular hospital admissions, with at most a 10% increase in admissions just north of San Francisco Bay. Since the same fire-originated $PM_{2.5}$ exposure surface is used for estimating the excess admissions for all three health outcomes, the concentration-response function (CRF) is what drives the differences in the percent increase in admissions. The largest fire-attributable increase in hospital admissions is observed for asthma admissions because it has a steeper CRF in comparison to respiratory and cardiovascular hospital admissions.

Text S5.

Figure S5 shows the total number of respiratory, cardiovascular, and asthma admissions attributable to fire-originated $PM_{2.5}$ in each county between 8-20 October 2017, estimated using base case assumptions. The counties that experienced the most fire-attributable hospital admissions are located in the Bay Area. This region experienced the greatest acute health impacts in part due to high population density and the extreme smoke concentrations estimated in these counties during the 2017 fires.

Text S6.

Figure S6 shows a comparison of the five health impact assessment approaches for estimating daily excess respiratory and cardiovascular admissions between 8-20 October. As shown, there is variety in both the magnitude and the temporal trends of the excess admissions estimated, due to differences in the smoke exposure surfaces and CRFs used. For example, while all five approaches capture 11 October as the day with the largest number of excess admissions, CC-CMAQ, compared to BME data fusion or BME kriging, predicts a much steeper drop in attributable admissions between 11-15 October. The non-wildfire (NF) CRF has the most impact on the temporal trends in excess admissions, with less respiratory and more cardiovascular admissions estimated across the entire fire period.

Figure S7 shows a comparison of the five health impact assessment approaches for estimating the rate of excess respiratory admissions across central California on 10 October. As shown, the assessment method used changes where high rates of excess admissions are located. When either CC-CMAQ or BME kriging is used to estimate total $PM_{2.5}$, instead of BME data fusion, there is an increase in the frequency of high excess admission rates and a shift in which regions are most impacted. Using the October 2016 surface to isolate fire-originated $PM_{2.5}$, instead of CMAQ percent attributable, does not notably impact the locations of high attributable admission rates, but does increase the number of regions in central California with an observable rate of excess admissions. There is minimal change in the locations of excess admission rates when the NF CRF is used.

Figure S8 shows a comparison of the four methods for estimating daily fire-originated $PM_{2.5}$ exposure. All of the exposure estimation methods produce notably different estimates of the spatial average, population-weighted average, and 95th percentile of wildfire $PM_{2.5}$, which explains some of the differences observed in the total number of excess admissions estimated. For example, using CC-CMAQ as the total exposure estimate results in higher estimated concentrations between 11-14 October compared to using BME data fusion or BME kriging. Further, using the October 2016 surface to isolate fire-originated concentrations, instead of CMAQ percent attributable, results in lower peaks in estimated concentrations on 11-12 and 16-17 October.

Figure S9 shows the sensitivity analysis comparing the 95% confidence intervals (CIs) of the estimated number of excess cardiovascular hospital admissions when uncertainty from either or both the CRF and total exposure surface are accounted for, for all three total $PM_{2.5}$ surfaces. As shown in the Results section, when uncertainty from both the CRF and total exposure estimate are accounted for, CC-CMAQ produces the largest CI around the cardiovascular admissions estimate and BME data fusion produces the smallest. For all three exposure surfaces, the CRF is the primary source of uncertainty in the final admissions estimate, but the relative amount of uncertainty contributed by the CRF varies widely. When CC-CMAQ is used, the CRF and total exposure estimate contribute approximately equal levels of uncertainty. In comparison,

when BME data fusion or BME kriging is used, the vast majority of uncertainty in the final estimate comes from the CRF, with the total exposure estimate contributing very little. Table S2 contains the numeric data of the uncertainty analyses presented in Figures 4 and S9.

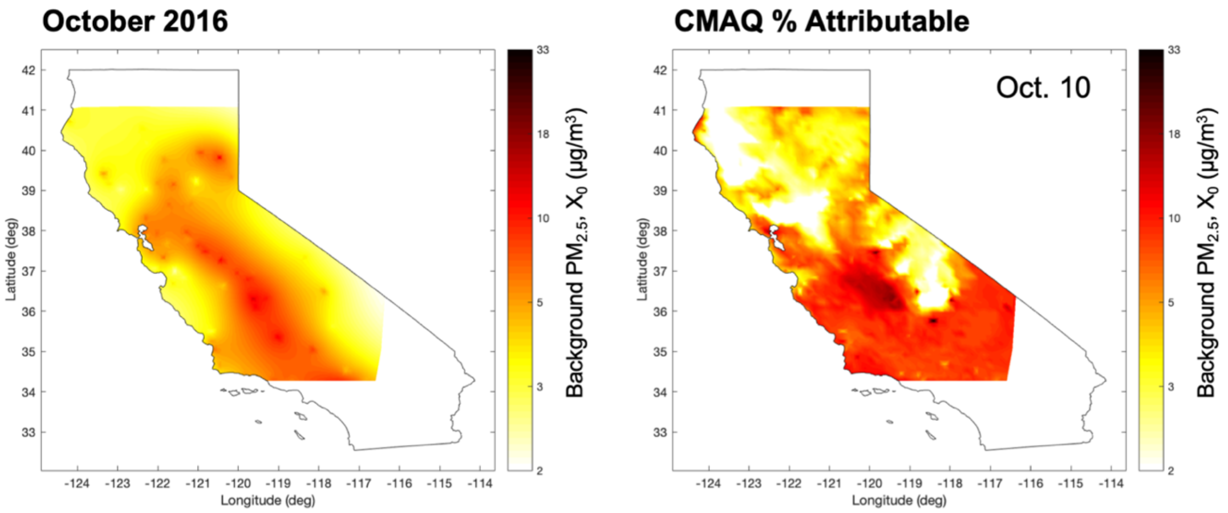

Figure S1. Comparison of methods for estimating background $PM_{2.5}$ concentrations during the October 2017 wildfires. Estimates obtained through BME kriging of October 2016 monitoring station observations (left) and percent of $PM_{2.5}$ attributable to background emissions from CMAQ output with and without fire emissions, shown on 10 October 2017 (right).

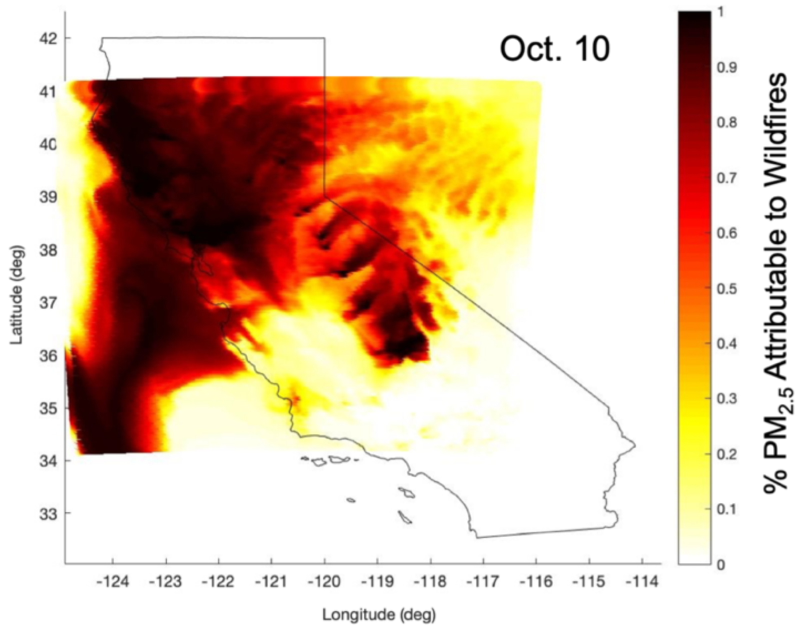

Figure S2. Estimate of the percent of $PM_{2.5}$ concentrations attributable to wildfire emissions on 10 October 2017, obtained from CMAQ output with and without fire emissions.

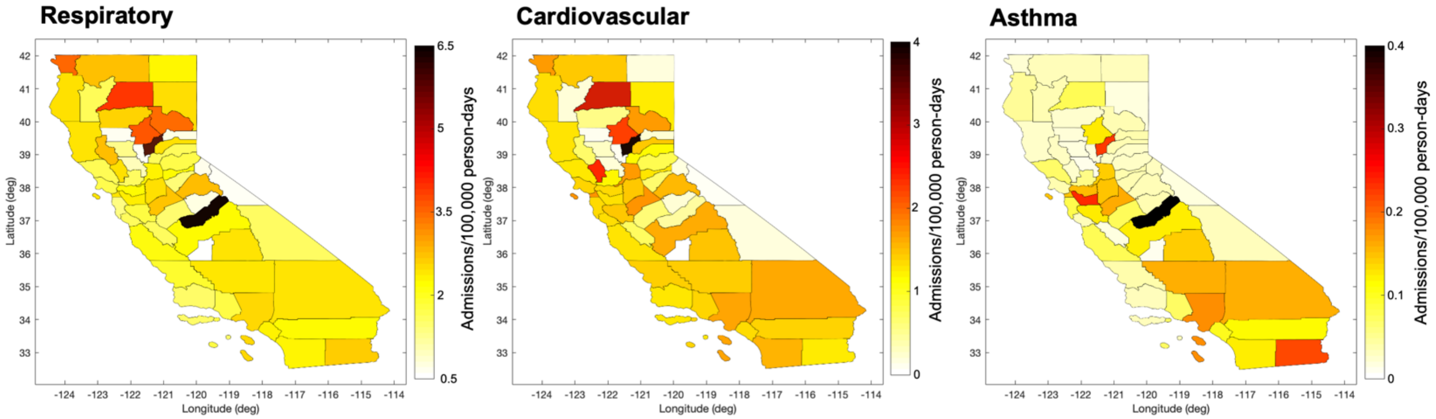

Figure S3. County-level background daily rate of respiratory, cardiovascular, and asthma hospital admissions across California in 2017.

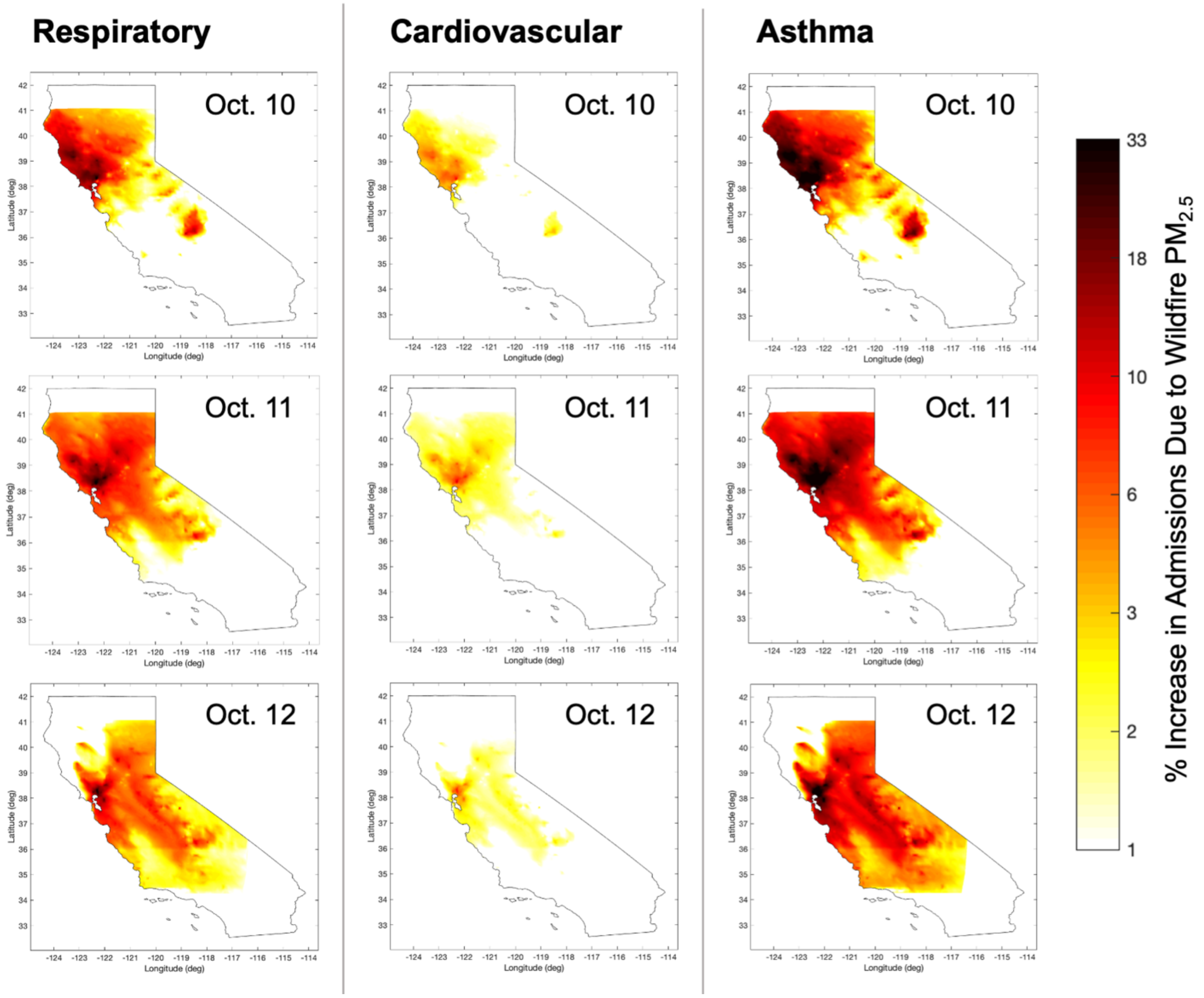

Figure S4. Percent increase in respiratory, cardiovascular, and asthma hospital admissions due to wildfire $PM_{2.5}$ exposure, 10-12 October 2017, estimated using base case assumptions.

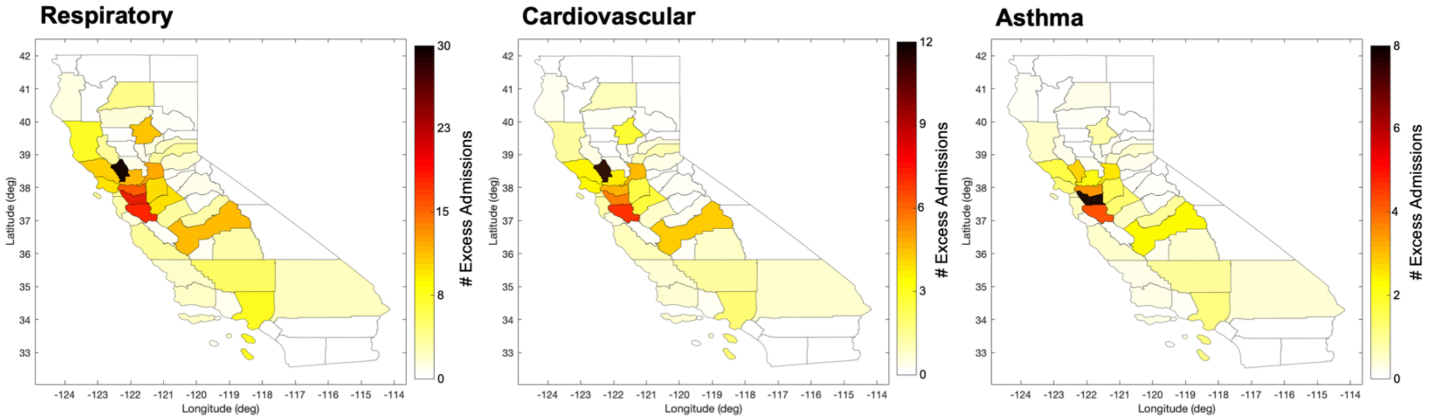

Figure S5. Number of excess respiratory, cardiovascular, and asthma admissions in each county, 8-20 October 2017, estimated using base case assumptions.

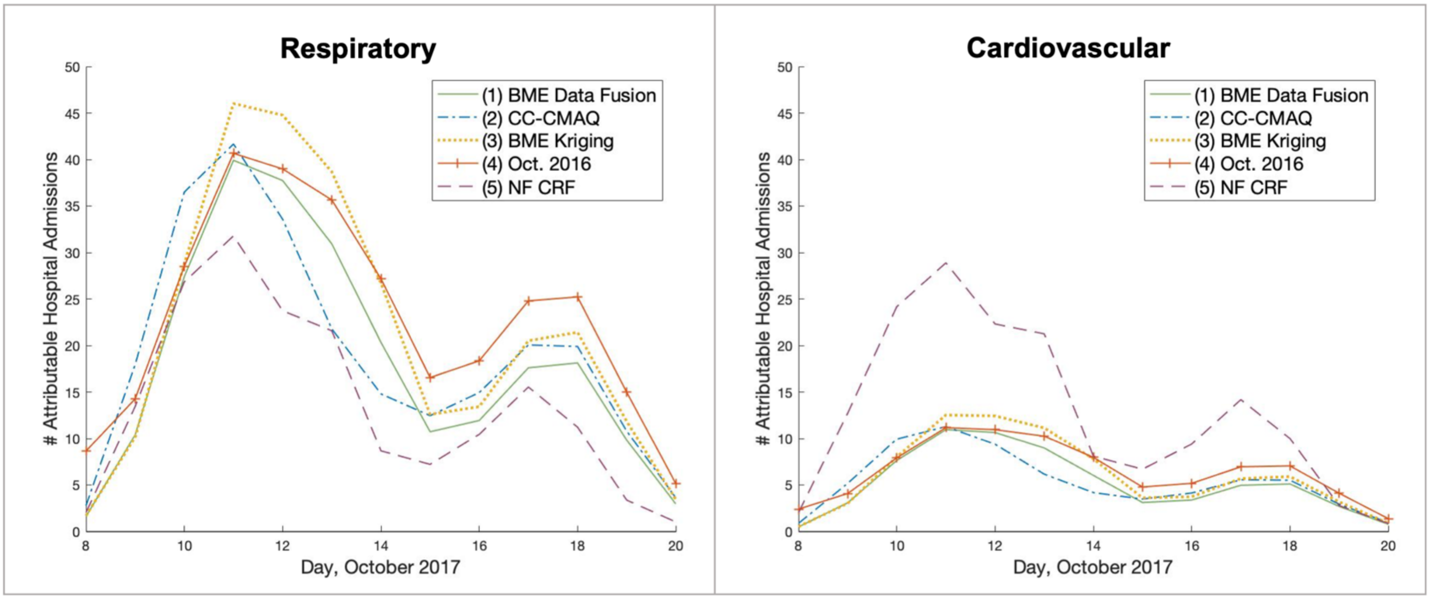

Figure S6. Comparison of the five health impact assessment approaches for estimating daily excess respiratory and cardiovascular hospital admissions between 8-20 October 2017. (1) Base case, BME data fusion with CMAQ % attributable and WF CRF; (2) CC-CMAQ with CMAQ % attributable and WF CRF; (3) BME kriging with CMAQ % attributable and WF CRF; (4) BME data fusion with October 2016 and WF CRF; (5) BME data fusion with CMAQ % attributable and NF CRF.

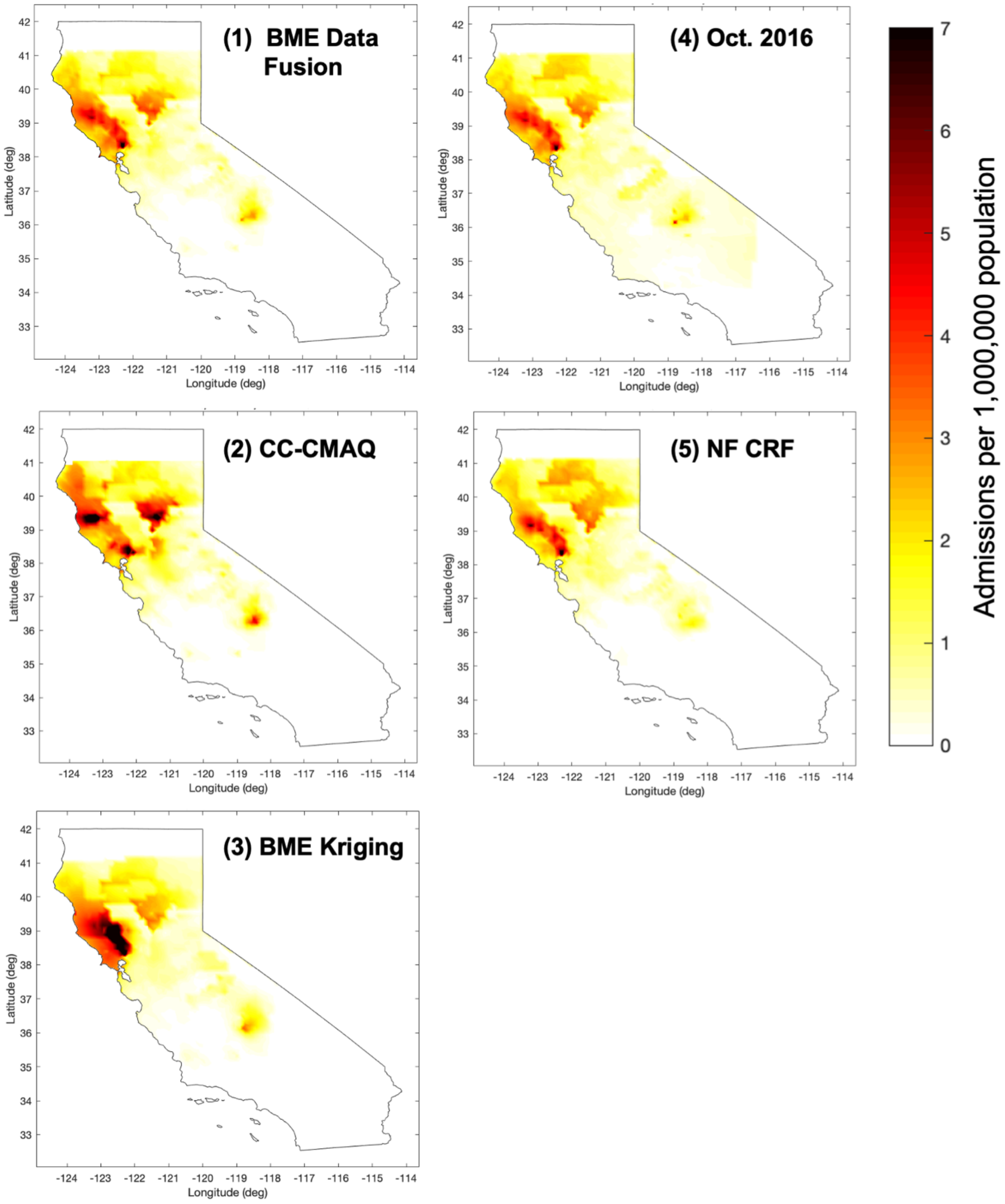

Figure S7. Comparison of the five health impact assessment approaches for estimating daily excess respiratory hospital admissions across central California on 10 October 2017. (1) Base case, BME data fusion with CMAQ % attributable and WF CRF; (2) CC-CMAQ with CMAQ % attributable and WF CRF; (3) BME kriging with CMAQ % attributable and WF CRF; (4) BME data fusion with October 2016 and WF CRF; (5) BME data fusion with CMAQ % attributable and NF CRF.

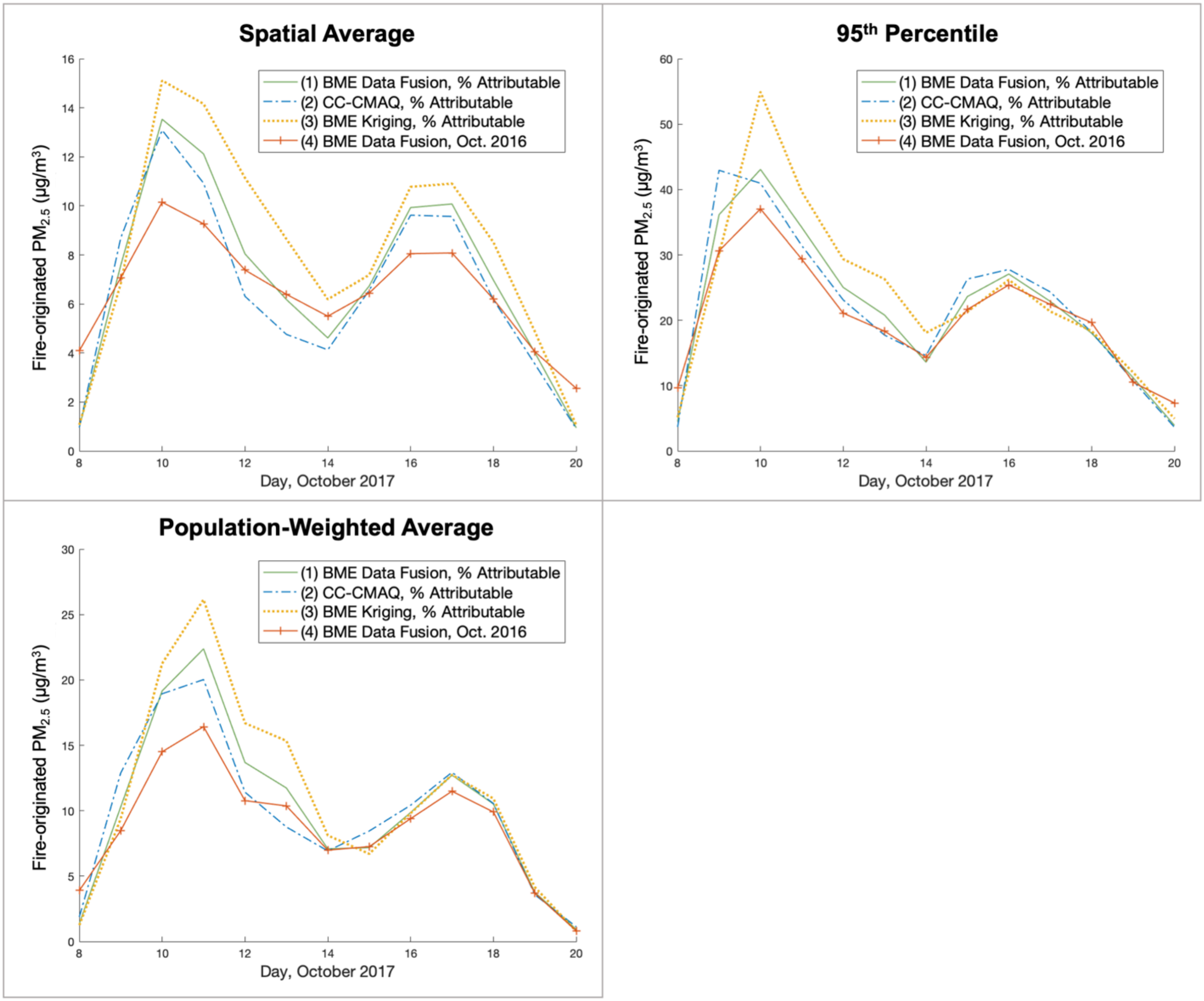

Figure S8. Comparison of the four methods for estimating exposure to fire-originated $PM_{2.5}$ between 8-20 October 2017. (1) Base case, BME data fusion with CMAQ % attributable as background; (2) CC-CMAQ with CMAQ % attributable as background; (3) BME kriging with CMAQ % attributable as background; (4) BME data fusion with October 2016 as background.

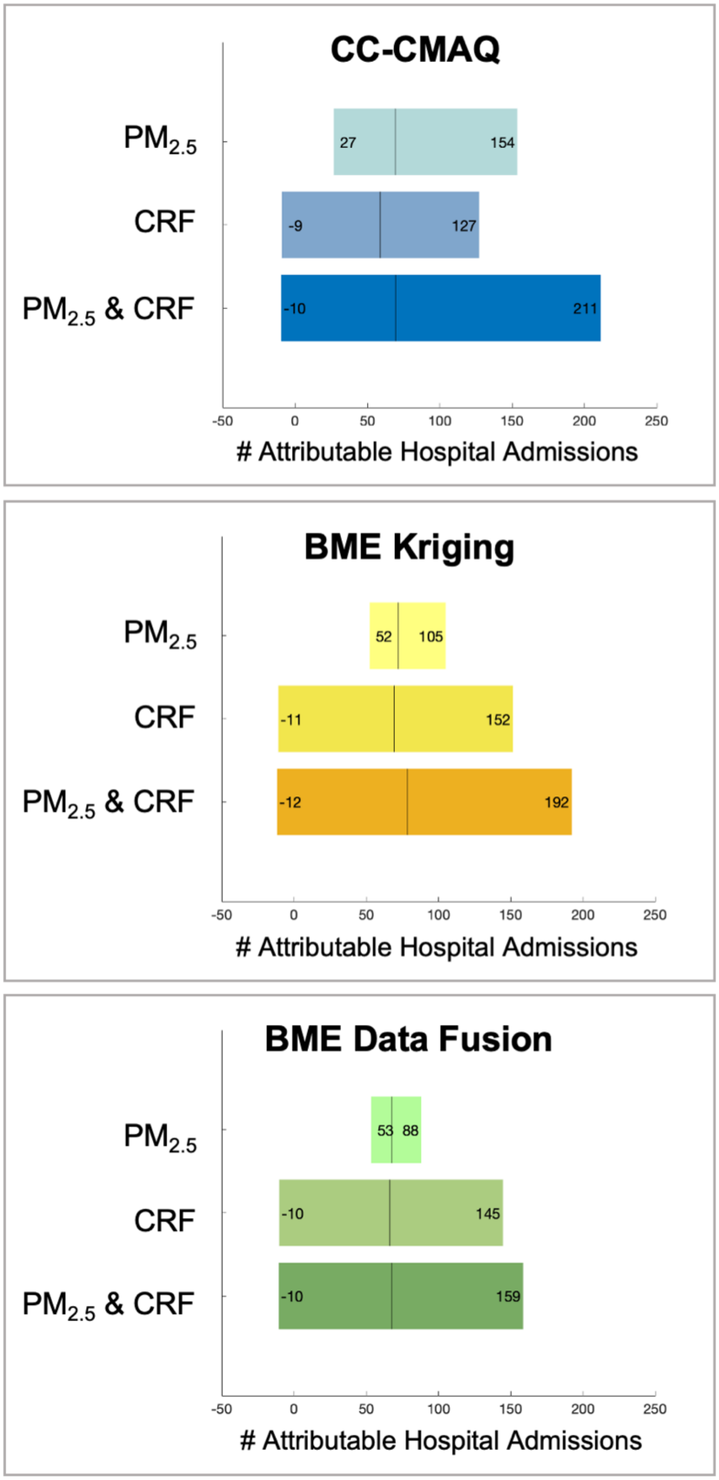

Figure S9. The individual contributions of uncertainties in the CRF and total $PM_{2.5}$ surface to the total uncertainty in estimated cardiovascular hospital admissions when uncertainties in both the CRF and total $PM_{2.5}$ surface are accounted for. Uncertainties are shown as 95% confidence intervals with the vertical line marking the mean estimate. Results are shown using the three total $PM_{2.5}$ exposure estimates (CC-CMAQ, BME kriging, and BME data fusion), which all use CMAQ % attributable for the background concentrations and the WF CRF.

Table S1. Number of daily excess respiratory, cardiovascular, and asthma hospital admissions attributable to wildfire-originated $PM_{2.5}$, 8-20 October 2017, estimated using base case assumptions.

**Date** *October 2017*	**# Attributable Admissions (95% CI)**		
	Respiratory	*Cardiovascular*	*Asthma*
8	1.61 (0.78, 2.69)	0.50 (-0.08, 1.15)	0.29 (0.12, 0.51)
9	10.40 (4.75, 18.32)	3.08 (-0.46, 7.40)	1.82 (0.71, 3.39)
10	27.42 (12.93, 46.47)	7.63 (-1.13, 17.86)	4.58 (1.8, 8.34)
11	39.91 (18.83, 67.76)	10.96 (-1.65, 25.75)	7.15 (2.81, 13.04)
12	37.74 (17.56, 64.54)	10.65 (-1.73, 25.12)	7.69 (2.98, 14.03)
13	30.94 (14.78, 51.46)	8.97 (-1.39, 20.84)	6.39 (2.53, 11.45)
14	20.33 (9.83, 33.61)	6.03 (-0.89, 13.94)	3.94 (1.6, 6.96)
15	10.73 (5.19, 17.85)	3.11 (-0.46, 7.23)	1.92 (0.79, 3.39)
16	11.94 (5.74, 19.87)	3.38 (-0.55, 7.86)	2.08 (0.85, 3.68)
17	17.60 (8.49, 29.25)	4.97 (-0.76, 11.50)	3.29 (1.34, 5.79)
18	18.13 (8.67, 30.19)	5.10 (-0.83, 11.79)	3.41 (1.38, 6.01)
19	9.82 (4.59, 16.88)	2.69 (-0.43, 6.39)	1.78 (0.71, 3.22)
20	2.96 (1.39, 5.08)	0.77 (-0.12, 1.83)	0.52 (0.21, 0.92)
**Total**	239.53 (113.54, 403.99)	67.84 (-10.48, 158.64)	44.85 (17.81, 80.73)

Table S2. Numeric data for the sensitivity analyses presented in Figures 4 and S9. All approaches use CMAQ % attributable as background concentration and the WF CRF.

Total $PM_{2.5}$ Exposure Estimate	Source(s) of Uncertainty	# Attributable Admissions (95% CI)	
		Respiratory	*Cardiovascular*
CC-CMAQ	Total $PM_{2.5}$	250 (95, 570)	69 (27, 154)
	CRF	210 (110, 310)	59 (-9, 127)
	Total $PM_{2.5}$ & CRF	251 (77, 620)	70 (-10, 211)
BME Kriging	Total $PM_{2.5}$	258 (182, 388)	72 (52, 105)
	CRF	246 (128, 367)	69 (-11, 152)
	Total $PM_{2.5}$ & CRF	280 (124, 512)	78 (-12, 192)
BME Data Fusion	Total $PM_{2.5}$	239 (186, 317)	68 (53, 88)
	CRF	233 (122, 348)	66 (-10, 145)
	Total $PM_{2.5}$ & CRF	240 (114, 404)	68 (-10, 159)
